# Multimaterial 3D Printing in Activating Bath Enables In Situ Polymerization of Thermosets with Intricate Geometries and Diverse Elastic Behaviors

**DOI:** 10.1002/adma.202508568

**Published:** 2025-08-13

**Authors:** Young Bum Lee, Yun Seong Kim, Chen Chen, Mohammad Tanver Hossain, Benjamin A. Suslick, Randy H. Ewoldt, Sameh H. Tawfick, Jeffrey S. Moore, Nancy R. Sottos, Paul V. Braun

**Affiliations:** ^1^ Beckman Institute for Advanced Science and Technology University of Illinois Urbana‐Champaign Urbana IL 61801 USA; ^2^ Materials Research Laboratory University of Illinois Urbana‐Champaign Urbana IL 61801 USA; ^3^ Department of Materials Science and Engineering University of Illinois Urbana‐Champaign Urbana IL 61801 USA; ^4^ Department of Mechanical Science and Engineering University of Illinois Urbana‐Champaign Urbana IL 61801 USA; ^5^ Grainger College of Engineering University of Illinois Urbana‐Champaign Urbana IL 61801 USA; ^6^ Department of Chemistry University of Illinois Urbana‐Champaign Urbana IL 61801 USA

**Keywords:** chemical activation, elastomer, embedded 3D printing, ring opening metathesis polymerization, thermoset

## Abstract

Polydicyclopentadiene, p(DCPD), is a high‐performance thermoset valued for its exceptional toughness, strength, and stiffness. When copolymerized with 1,5‐cyclooctadiene (COD), its mechanical properties can be tuned from glassy to rubbery at room temperature. While frontal polymerization enables a rapid and energy‐efficient route to 3D print DCPD‐based materials, challenges such as ink shelf life and gravitational distortion, especially in direct ink writing of soft COD‐rich formulations, must be considered. Here, a complementary chemical strategy is presented, embedded 3D printing, that enables localized in situ polymerization of printed DCPD/COD inks within a reactive support matrix. The matrix provides both physical support and a reservoir of chemical activator, which diffuses into the ink, activates a latent bis(N‐heterocyclic carbene) Ru precatalyst, and initiates ring‐opening metathesis polymerization. Curing begins at the ink–matrix interface and propagates inward via diffusion, stabilizing the interface and preventing capillary‐driven deformation regardless of the matrix yield stress. This approach eliminates the need for cold storage, external curing, or photoinitiation, significantly expanding the processing window. Using this method, diverse thermosetting and elastomeric architectures are fabricated with features as small as 5 µm and aspect ratios of 100, including interlinked chains, shallow spherical shells exhibiting snap‐through buckling, and hair‐like fin arrays inaccessible through traditional techniques.

## Graphical abstract

A new embedded 3D printing (EMB3D) strategy enables in situ polymerization of DCPD/COD‐based inks within a fugitive reactive matrix where the activator in the matrix cures the printed filament. Cured ink properties are tunable through DCPD‐COD comonomer ratios, yielding materials from stiff thermosets to soft elastomers. This approach allows fabrication of complex 3D structures containing high‐aspect‐ratio features as small as 5 µm.

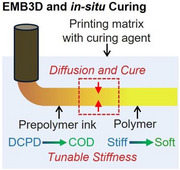

## 1 Introduction

Custom and rapid fabrication of high‐fidelity structures with complex geometries requires new manufacturing approaches.^[^ ^1^, ^2^ ^]^ For example, typical silicone and epoxy‐based materials used in the aerospace, automotive, and energy industries are manufactured with slow, high‐temperature curing processes (e.g., compression molding or reactive injection molding).^[^ ^3^, ^4^, ^5^, ^6^, ^7^ ^]^ These existing technologies rely on a preexisting structural mold to determine the polymer shape. Additionally, many molding‐based processes struggle to produce intricate geometries of small sizes since the removal of the supporting framework can damage the final structure. These needs created a market for new, robust 3D printing technologies.^[^ ^8^, ^9^, ^10^, ^11^ ^]^ In contrast to molding processes, 3D printing serially fabricates structures in real time through a variety of strategies. For example, digital light processing (DLP),^[^ ^12^, ^13^ ^]^ stereolithography (SLA),^[^ ^14^ ^]^ and direct ink writing (DIW)^[^ ^15^, ^16^, ^17^ ^]^ successfully print structural thermosets using a cure process to convert a prepolymer into the final material. Extrusion‐based DIW printing, which involves a layer‐by‐layer deposition process of extrudable materials, is particularly flexible in that it accommodates the production of a wide array of materials^[^ ^18^ ^]^ (e.g., metals, ceramics, polymers, biomaterials). With DIW, inks can contain well‐dispersed additives^[^ ^17^, ^19^ ^]^ (e.g., carbon fibers, magnetic nanoparticles, or metal nanowires) to impart unique properties and mechanical characteristics to the printed object. The ability to manipulate the ink composition facilitates the creation of customized, functionally graded, and hierarchical structures.^[^ ^20^, ^21^ ^]^

DIW can encounter challenges, particularly when writing low‐viscosity inks or soft materials prone to structural collapse.^[^ ^1^ ^]^ Gravitational forces influence the printing process and cause sagging, pinching, spreading, shifting, or distortion. A common approach to mitigate these effects involves conversion of low‐viscosity thermosetting inks into yield‐stress fluids by incorporating rheological modifiers (e.g., micron‐scale particles, nanosized silica, emulsions, waxes).^[^ ^22^ ^]^ This modification, however, alters the properties of the printed structure and requires laborious optimization to adjust the ink composition and characteristics for effective printing. Alternatively, in situ gelation of the extruded ink at the nozzle tip, followed by rapid curing, can minimize gravity‐driven effects.^[^ ^3^ ^]^ Such gelation strategies, facilitated by methods like frontal polymerization^[^ ^23^, ^24^ ^]^ or photo‐curing,^[^ ^25^ ^]^ support the solidification of stiff thermoset materials of specific chemistries. Challenges persist in printing structures that integrate gradients of soft and hard materials or have large aspect ratios. Such structures may fail even after curing if they cannot support their own weight or that of subsequent layers. This underscores the necessity for support and curing methodologies to enhance the capabilities of 3D printing for handling diverse material properties and complex, multi‐material geometries.

Embedded 3D printing (EMB3D), in contrast, circumvents gravity‐driven challenges through the use of viscosity‐modified support baths.^[^ ^26^, ^27^, ^28^, ^29^, ^30^ ^]^ The bath supports the shape of freshly extruded liquid inks without the need for auxiliary supports.^[^ ^16^, ^31^, ^32^ ^]^ This technique significantly broadens the scope of accessible materials, such as silicones, rubbers, and fibers.^[^ ^29^ ^]^ EMB3D also facilitates the preparation of composite materials comprised of unconnected particles, nanomaterials, and cells, with applications to catalysis, optics, mechanics, or drug delivery.^[^ ^33^ ^]^

Despite these advantages, existing EMB3D methods introduce different complexities to consider. With the printing step and curing step being temporally separated, surface tension or writing stresses can impact the integrity of the printed structure. For example, surface tension‐induced pinching or interactions between the ink and the matrix can disconnect or distort shapes.^[^ ^34^ ^]^ Cure stimuli (e.g., heat, light) can introduce variability into the printed structure if they affect the physical properties of the bath and ink (e.g., viscosity, surface tension).^[^ ^3^ ^]^ Light‐based cures are limited to the light penetration depth into the bath containing the written structure and require a reasonable polymerization quantum yield.^[^ ^34^ ^]^ A chemical diffusion‐based curing approach has been reported,^[^ ^35^ ^]^ where a sacrificial ink is used to create vascular structures. Approaches involving reactions initiated at liquid–liquid interfaces between the ink and matrix and small molecule diffusion between the ink and matrix are underexplored.

Here we demonstrate embedded 3D printing of a prepolymer ink containing a masked precatalyst within an activating matrix. Inspired by biological systems where interfaces are maintained while small molecules selectively diffuse (e.g., blood vessels), our approach utilizes an immiscible ink and matrix system where the curing agent diffuses from the matrix and into the ink to trigger the curing reaction. A bis(*N*‐heterocyclic carbene) Ru complex^[^ ^36^, ^37^ ^]^ is employed as a latent ROMP catalyst in the ink. This precatalyst remains inactive in the absence of an activator, even when dissolved in highly strained cyclic olefins, such as dicyclopentadiene (DCPD) or 1,5‐cyclooctadiene (COD). A Cu(I) activator dispersed into the matrix triggers polymerization immediately upon ink deposition to create a solid polymer interface that over time cures through the thickness of the ink (up to a diffusion limit). The interfacial polymer skin rapidly fixes the printed shape against surface tension‐induced pinching, even across a range of matrix surface tensions and yield stresses. The ink zero‐shear viscosity was carefully matched to that of the matrix by pre‐polymerization, eliminating the need for rheological modifiers that could otherwise compromise the properties of the printed layer. The structures fabricated by EMB3D exhibited mechanical and thermochemical properties determined by the monomer composition of the ink. As a demonstration, we fabricated complex structures of a variety of geometries, including interlocking chain‐links, shallow spherical shells, and hair‐like flexible brushes.

## 2 Results and Discussion

### 2.1 Liquid–Liquid Interfaces and Chemical Activation in EMB3D

**Figure 1:**
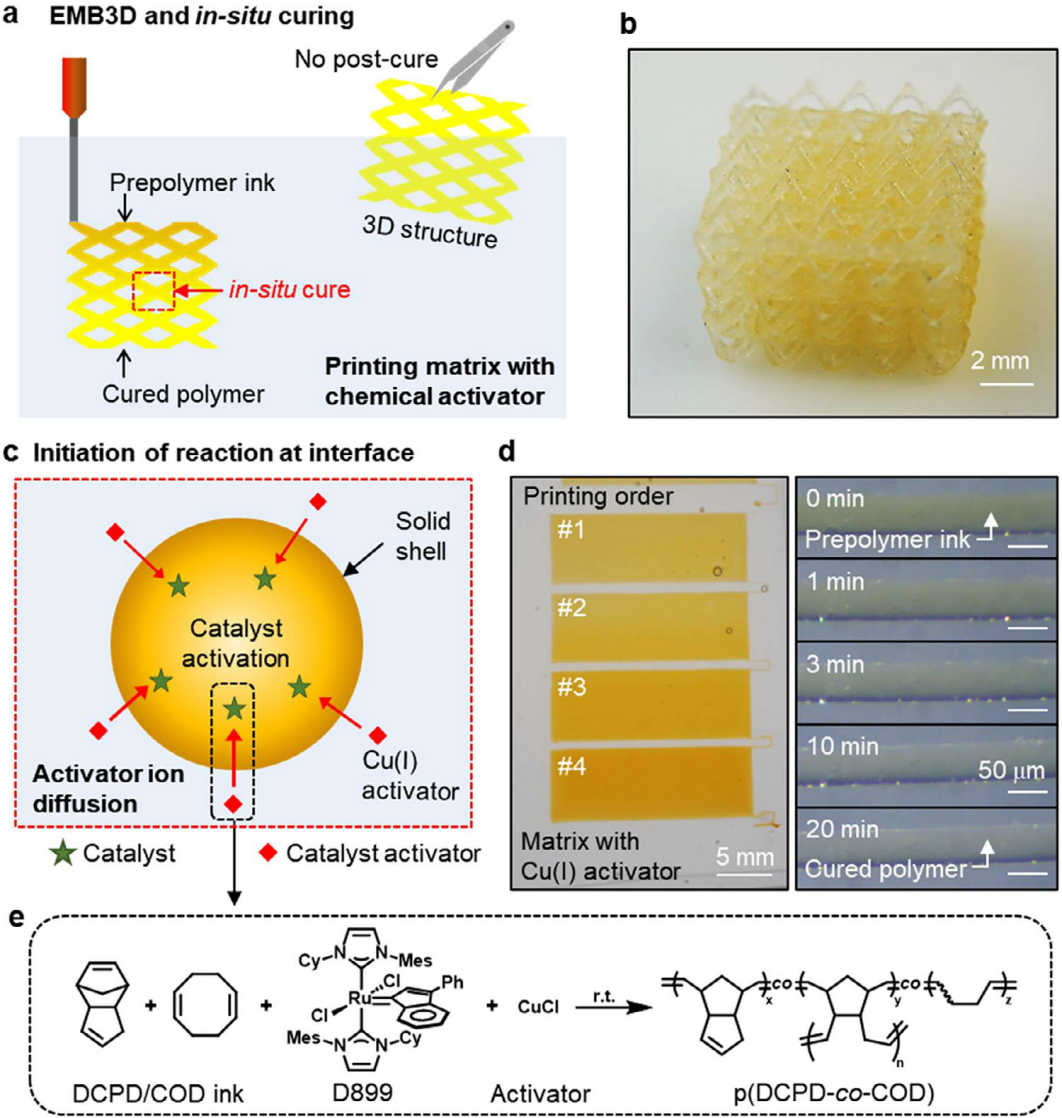
EMB3D in chemical curing matrix. a) Printing and in situ curing process in a matrix containing a chemical activator. b) Printed octahedral truss structures spontaneously cured in the matrix. c) Diffusion of activator ions from the matrix to the ink, chemically curing the prepolymer ink. d) Four sequentially printed rectangles (20 mm × 7 mm), each requiring 5 min to print (left), and optical microscope images of the filament curing process (right). e) Polymerization of DCPD/COD ink through the activation of D899 by a Cu(I) ion.

**Figure** **1** illustrates the concept of EMB3D within a chemical curing matrix, where extruded DCPD or COD ink is supported and cured. The resin ink contains all formulation components except one chemical (i.e., initiator or activator), which triggers resin curing upon diffusion of activating chemicals. By compartmentalizing reactive compounds, the ink pot life is significantly over three months, thereby obviating the need for cold storage due to the ink residual reactivity.^[^ ^36^ ^]^ Upon printing inside the chemical curing matrix, polymerization initiates spontaneously as the chemical activator diffuses radially from the matrix into the ink solution through the interface, facilitating room‐temperature curing within minutes without external triggers or post‐curing processes such as heating (Figure 1a). For example, an octahedral lattice structure fabricated using DCPD ink (Figure 1b) produces uniform lattice nodes without additional curing steps. Each line in the lattice is formed by filaments with a diameter of ≈200 µm, resulting in robust junctions enhanced by the incorporation of antioxidants to improve interlayer bond strength.^[^ ^37^ ^]^

In EMB3D, a liquid–liquid interface initially forms between the printing ink and the embedding matrix. In systems where components freely diffuse, instability at the liquid–liquid interface may lead to intermixing, irregular boundaries, and dimensional variations in the printed structures. To prevent unintended mixing, a printing formulation that maintained a distinct liquid–liquid interface while facilitating diffusion of chemical activators was developed. This approach stabilizes the interface to ensure the structural integrity and fidelity of printed constructs, allowing time for cure reactions to initiate. During printing, the extruded ink makes initial contact with the matrix solution. Depending on their mutual solubilities—the extent to which two substances can dissolve in each other—the ink and matrix solutions may mix or remain phase separated.^[^ ^38^, ^39^ ^]^ The printing formulation (see Section 2.2) was engineered to prevent intermixing, thereby establishing a distinct interface.

Subsequently, chemical activators in the matrix diffuse across the interface, initiating polymerization (Figure 1c). Here we employed a masked bis(*N*‐heterocyclic carbene) ruthenium precatalyst, D899, that supports frontal polymerization (FP)^[^ ^24^, ^31^, ^36^ ^]^ and ring‐opening metathesis polymerization (ROMP) upon activation.^[^ ^37^ ^]^ At the ink‐matrix interface, Cu(I) diffuses into the ink and activates the catalyst, initiating polymerization to form an outer solid layer. Activator diffusion throughout the printed filament induces curing of the entire resin, provided that Cu(I) can diffuse to the center of the filament. Curing of the resin can be tracked by color change (Figure 1d, left), where the uncured resin is orange, and fully cured p(DCPD) appears as a transparent yellow due to the presence of antioxidants. Optical microscope images reveal negligible shape deformations and volume changes during the curing process, and the color transition completes after 20 min (Figure 1d, right). Activated catalysts polymerize monomer or co‐monomer inks (e.g., DCPD or COD) into crosslinked p(DCPD) thermosets, soft p(COD) rubbers, or their copolymers (Figure 1e). The filament diameters were maintained below the diffusion length limit to ensure complete polymerization of the resin (see Section 2.3).

### 2.2 Matrix Effects on EMB3D Quality

**Figure 2:**
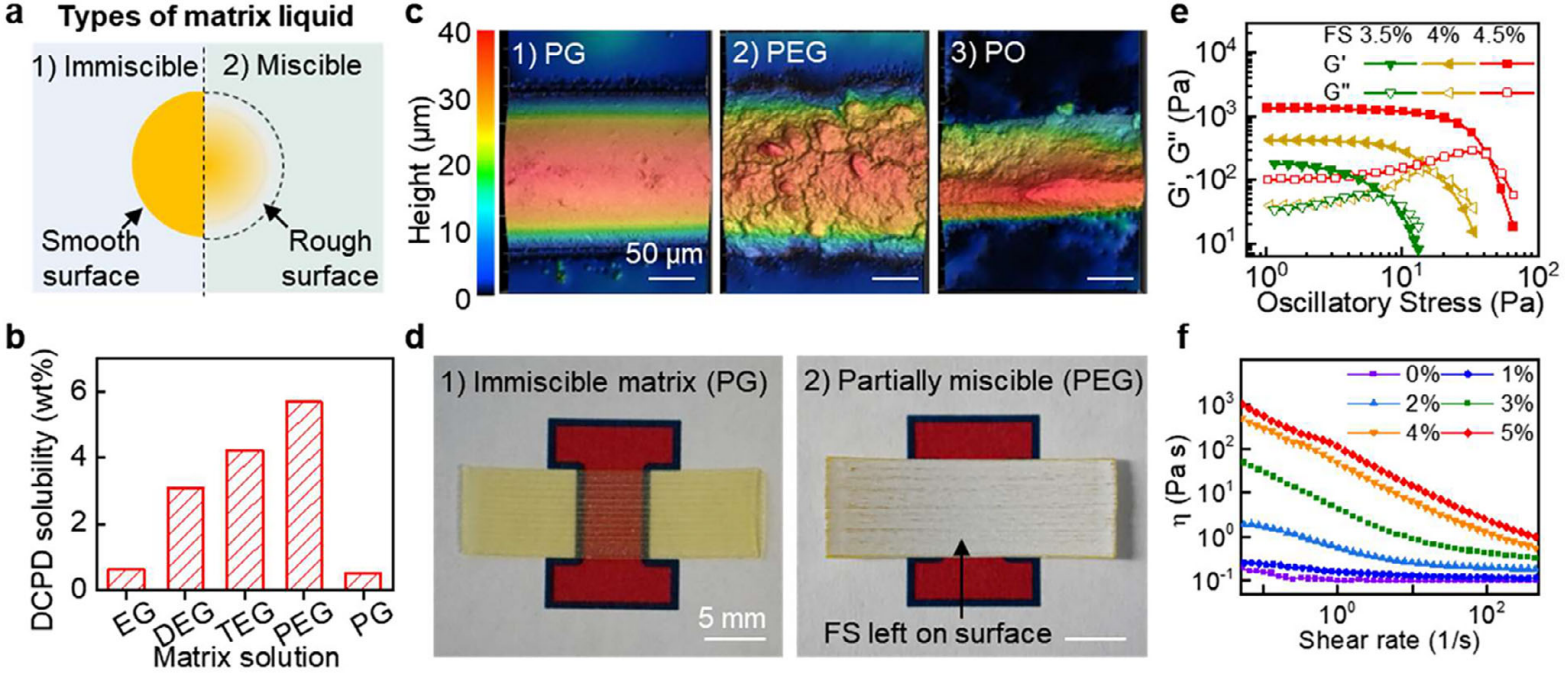
Effect of matrix chemistry on EMB3D. a) Illustration of the effect of miscibility on surface topology. b) Solubility of DCPD in different glycol solutions: ethylene glycol (EG), diethylene glycol (DEG), triethylene glycol (TEG), polyethylene glycol (PEG), and propylene glycol (PG). c) Surface topology of threads printed in 1) immiscible (PG), 2) partially miscible (PEG), and 3) fully miscible (PO) matrices. d) Sample images printed in PG and PEG matrix solutions (FS is fumed silica). e,f) Rheological properties of an immiscible matrix solution containing FS using (e) large amplitude oscillatory shear (LAOS) test and (f) shear rate sweep test.

EMB3D relies on selecting an appropriate matrix liquid and tuning its properties to mitigate surface instability caused by inter‐miscibility or surface tension between the ink and matrix (**Figure** **2**a). We evaluated three categories of matrix materials: 1) immiscible, 2) partially miscible, and 3) totally miscible with the ink solution. The mutual solubility values of nine matrix solutions were evaluated (Figure 2b; Table S1, Supporting Information), with the solubility of each phase in the other measured using NMR spectroscopy (Figures S1 and S2, Supporting Information). The chemical nature of the matrix solution determines its compatibility with the ink (here using DCPD as an ink stand‐in). Hydrocarbons (e.g., paraffin oil, PO) and monohydric alcohols (e.g., isopropyl alcohol) are completely miscible with DCPD. Hydrophobic diol liquids (e.g., polypropylene glycol) also exhibit complete miscibility, while hydrophilic diols (e.g., polyethylene glycol, PEG) phase separate from DCPD. Despite phase separation, the solubility of higher molecular weight diols (e.g., diethylene glycol (DEG), triethylene glycol (TEG), PEG) in DCPD increases with diol molecular weight; thus, we categorize these diols as partially soluble. Lower molecular weight diols such as propylene glycol (PG) or ethylene glycol (EG) exhibit low solubility in DCPD (below 0.6 wt%) and are considered immiscible matrix solutions.

Surface topography of threads printed in immiscible, partially miscible, and miscible matrices was characterized using profilometry (Figure 2c; Figure S3, Supporting Information). Threads printed in immiscible liquids such as EG or PG exhibit sharp liquid–liquid interfaces and yield smooth polymer surfaces after curing. In contrast, partially miscible matrices, such as PEG, while roughly maintaining filament dimensions, exhibit blurred or rough interfaces. Notably, polymers printed within a PEG matrix possessed a white and turbid surface (Figure 2d), whereas those in a PG matrix remained transparent. Rheology modifiers (e.g., fumed silica, FS) in the matrix blend into the ink‐matrix interface and adhere to the printed surface, contributing to the white appearance (Figure S4, Supporting Information). Finally, for fully miscible systems, such as with PO, monomers from the ink diffuse extensively into the matrix, leading to polymer threads with reduced diameters.

The rheological properties of the immiscible matrix are tuned by incorporating commercially available hydrophobic FS. Increasing silica concentration enhances matrix viscosity, imparting a yield stress fluid behavior (Figure 2e; Figure S5, Supporting Information) with shear thinning characteristics (Figure 2f). The zero‐shear viscosity (η_0_) and yield stress (σ_*y*_) can be adjusted within a range of 10^−1^ to 10^3^ Pa s and 7 to 51 Pa, respectively, by varying the additive loading from 1 to 5 wt%. The EG matrix formed a turbid solution when a hydrophobic rheology modifier was added due to its hydrophilicity, prompting the use of PG as the preferred printing matrix.

### 2.3 Activator Effect on Printing and Curing

**Figure 3:**
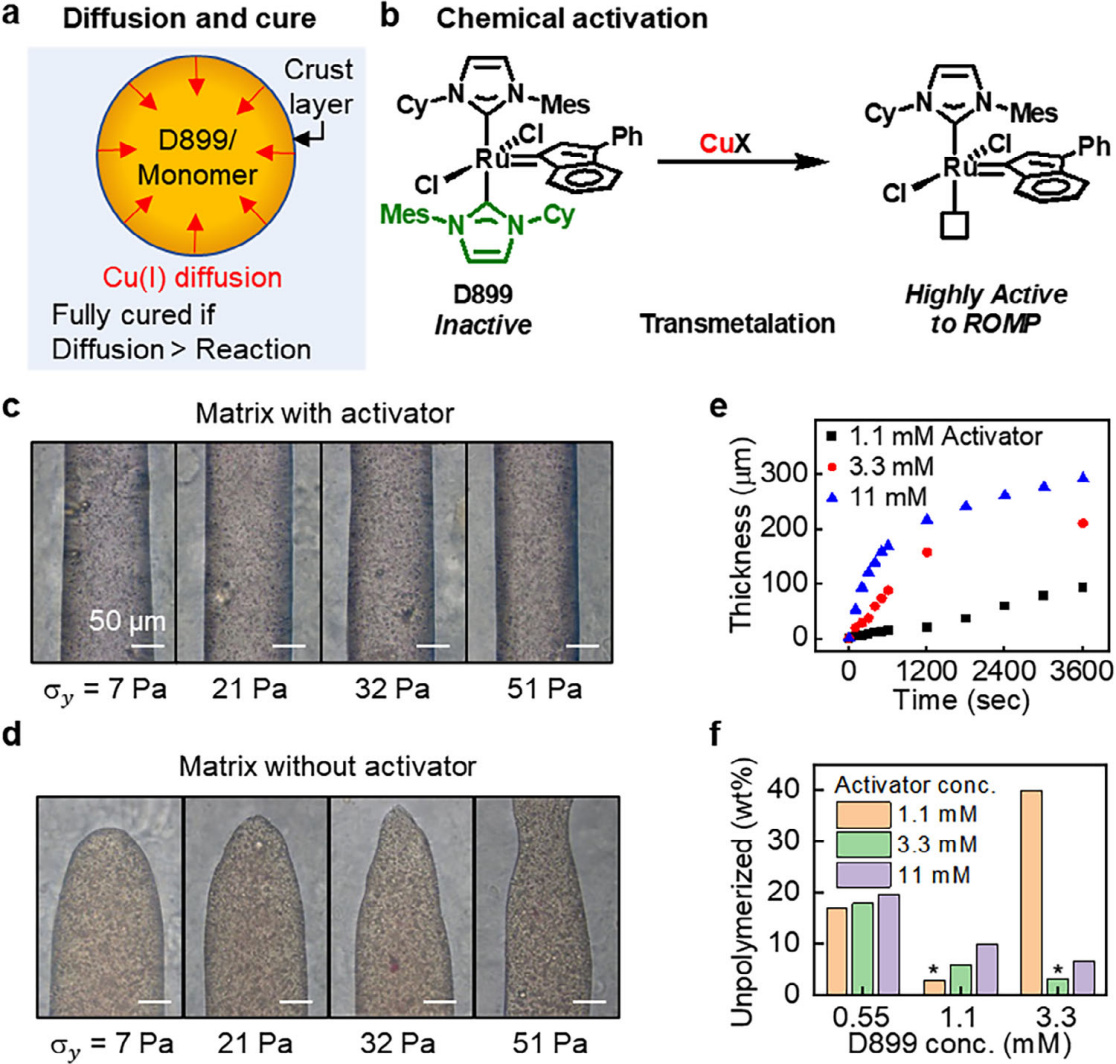
Chemical curing matrix formulation and its effect on EMB3D. a) Illustration showing the curing process of the printed ink in a chemical curing matrix. b) Activation of D899 with a Cu(I) ion yields a highly active ROMP catalyst via NHC transmetalation for polymerizing DCPD and COD. c,d) Effect of the activator on the printing geometry. Printed features of liquid inks in the matrix with activator (c) and without activator (d). Yield stress (σ_y_) was controlled by varying the concentration of FS. e) Polymer layer thickness as a function of time at the interface for various activator concentrations. f) Effect of catalyst in the ink and activator in the matrix stoichiometry.

**Figure** **3** illustrates activator diffusion from the matrix and the effects of activator concentration and stoichiometry on the curing process. The diffusion‐triggered reaction initiates at the surface of the printed ink as the Cu(I) activator diffuses into the resin (Figure 3a). For sufficiently thin filaments, the printed ink is fully activated and polymerized. However, for filament diameters exceeding 400 µm, the core remains liquid due to limited activator diffusion (Figure S6, Supporting Information). In this study, unless otherwise noted, fully cured samples—defined by filament diameter below the diffusion limit and curing times of at least 20 min—are used. Filaments printed in partially miscible matrices (e.g., PEG) are also cured by activator diffusion but exhibit a white, turbid appearance at the interface shortly after printing (Figure S7 and Video S1, Supporting Information). Figure 3b shows the chemical activation mechanism of the D899 catalyst via NHC transmetalation with Cu(I) salts, which sequester one NHC ligand, thus freeing the ruthenium center to coordinate monomers and initiate polymerization. The active, 14‐electron metathesis complex effectively polymerizes DCPD and COD monomers. The catalyst remains dormant until transmetalation with Cu(I) opens a coordination site for the incoming monomer.^[^ ^40^, ^41^ ^]^ While a Cu(I)‐based activation strategy is effective and was the focus here, some applications, e.g., biomedical, require consideration of activator compatibility, toxicity, and stability. Other metal‐based activators such as Au(I) and Ag(I) can trigger similar transmetalation‐based activation.^[^ ^40^, ^42^ ^]^ Ag(I) is generally considered biocompatible at low concentrations, but may raise ecological concerns. Au(I) is relatively inert, but cost‐prohibitive for many uses. Proton‐mediated activation of Ru catalysts^[^ ^43^, ^44^ ^]^ or emerging metal‐free ROMP (MF‐ROMP) strategies,^[^ ^42^, ^45^ ^]^ e.g., using organic photoredox initiators, could offer alternative pathways to reduce metal burden and enhance biocompatibility and present opportunities for future EMB3D systems.

To observe the interfacial polymerization, we prepared bulk solutions with phase‐separated layers without a rheological modifier (Figure S8, Supporting Information). The upper organic layer contained DCPD and D899 catalyst, while the lower aqueous layer contained the Cu(I) chemical activator. Polymerization initiated at the phase‐separated interface to form a polymer layer ≈200 µm thick, which then stopped growing in thickness. Presumably, once the polymer was this thick, it sufficiently blocked the diffusion of the activator into the monomer. Consequently, the bulk monomer phase remained unreacted. In contrast, lower activity activators such as Cu(II) exhibited curing times exceeding 2 h, which allowed them to fully diffuse into the monomer before cure and then cure the bulk solution. While the use of such lower‐activity activators may enable the curing of thick printed structures due to the longer time the ink remains fluid, surface instabilities, such as distortion or potential adsorption of FS from the surrounding matrix, may occur. Consequently, such activators were not investigated further. To mitigate potential oxidation or disproportionation of Cu(I), ascorbic acid was included as a reducing agent in the bath formulation. The ascorbate maintains Cu(I) stability by continuously reducing any Cu(II) that forms.^[^ ^46^ ^]^ Additionally, the bath was stored in a nitrogen environment to minimize oxygen exposure. Under these conditions, the activator solution remained clear and catalytically active over a few months. When stored under ambient conditions, the solution remained effective for approximately three weeks. After ascorbic acid depletion, a gradual color shift to pale green indicated partial oxidation to Cu(II).

The effect of the Cu(I) curing agent on printing quality was investigated by comparing printed features in matrices with and without activator (Figure 3c,d). Matrices containing activator maintained cylindrical filament shapes, regardless of the matrix yield stress value. In contrast, matrices without an activator exhibited shape deformation due to surface tension, leading to filament separation. Filaments in activator‐free systems experienced breakage and thickening within 20 min in matrices with lower yield stress (σ_y_ = 7 Pa), whereas those printed with higher yield stress matrices (σ_y_ = 51 Pa) deformed to a lesser extent, which reflects the matrix resistance to ink flow. The rapidly formed interfacial polymer layer when Cu(I) is presented maintained the shape of the printed material and reduced susceptibility to disruption from external stresses throughout the printing process.

The rate of film growth was observed under an optical microscope, and the film thickness was estimated from the change in contrast, assuming a sharp concentration gradient. With increasing activator concentration, film growth accelerated significantly in the initial 10 min. After 20 min, the growth rate significantly decreased and eventually reached a quasi‐steady state (Figure 3e), consistent with our hypothesis that the polymer formed hinders activator diffusion to the filament core. Higher catalyst concentrations in the ink enhanced both the growth rate and final film thickness, as depicted in Figure S9 (Supporting Information). The diffusion coefficient of Cu(I) was calculated to be ≈6 × 10^−8^ cm^2^ s^−1^ based on the film thickness vs. time over 20 to 30 min.

The catalyst to activator stoichiometry significantly affects the resultant polymer properties, including the glass transition temperature (*T_g_*), as previously reported.^[^ ^36^ ^]^ This is partly because partially polymerized resin and volatile monomers can remain within the polymer matrix. The unpolymerized portion of the printed samples was estimated using thermogravimetric analysis (TGA), measuring mass loss at ≈200 °C (Figure S10, Supporting Information). When the catalyst and activator concentrations were at or near a stoichiometric ratio, a minimum mass loss of 3% was achieved (Figure 3f), likely corresponding to the solvent (phenylcyclohexane) used to dissolve the catalyst. In contrast, incomplete curing was evident at suboptimal conditions—such as 0.55 mm catalyst regardless of activator concentration or 1.1 mm activator with 3.3 mm catalyst for a 400 µm filament—resulting in greater mass loss. We note this system cured only at the interface, while the center of the structure remained liquid. This increased volatilization is attributed to the retention of residual monomer or oligomers in the uncured interior regions of the filament. Note, thermal treatment alone was ineffective at activating the latent catalyst to initiate ROMP. These results underscore the importance of maintaining an appropriate catalyst‐to‐activator ratio and sufficient activator diffusion to ensure complete and uniform curing of the printed structure.

### 2.4 Tuning Ink Viscosity Via Partial Polymerization

**Figure 4:**
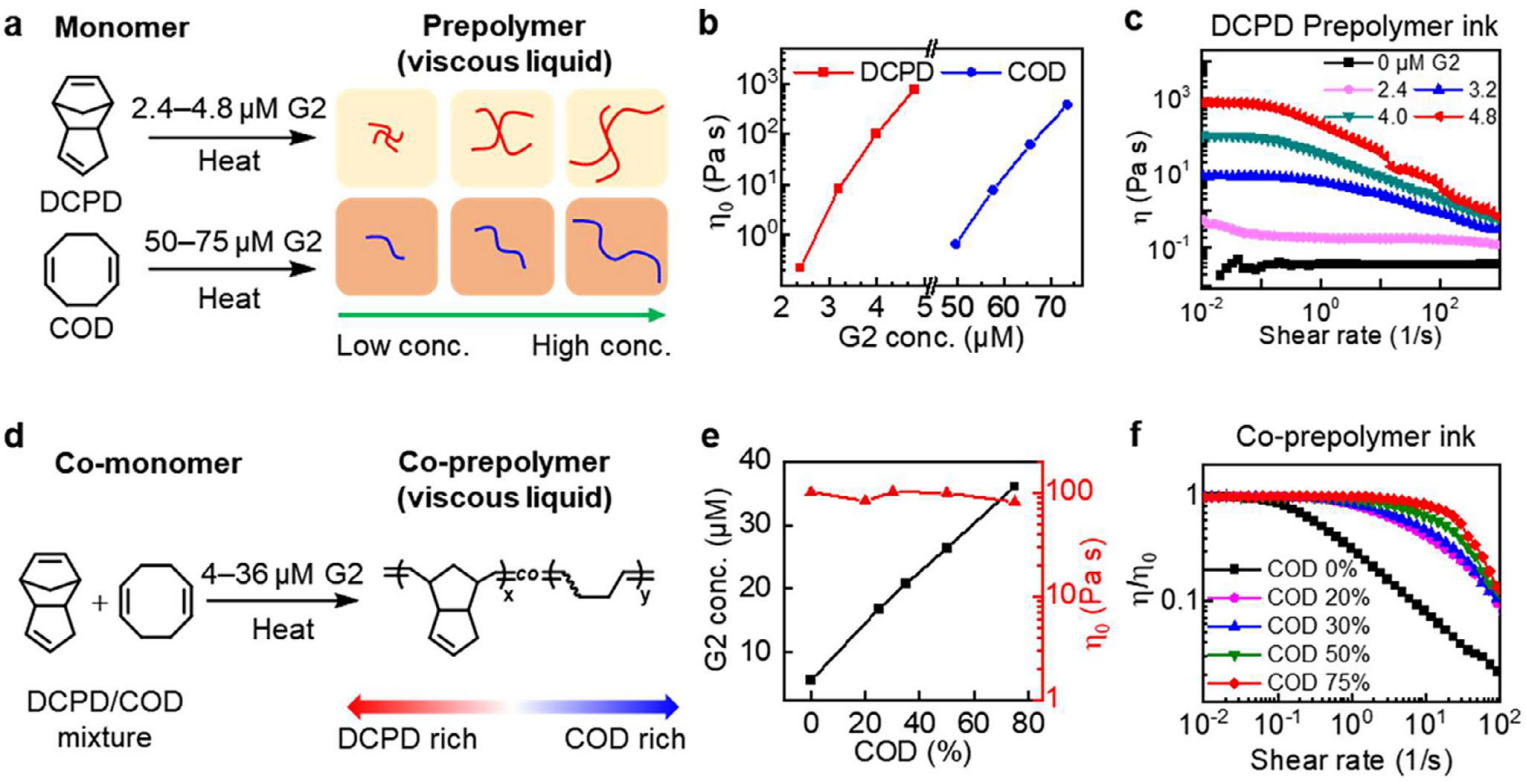
Tuning rheological properties of ink via partial polymerization. a) Schematic of partial polymerization for DCPD or COD monomers at various G2 concentrations. b) Zero‐shear viscosity of DCPD and COD solutions after viscosity modification. c) Viscosity and shear‐thinning behavior of DCPD prepolymer inks. d) Reaction scheme for partial co‐polymerization of DCPD and COD monomer mixtures. e) Proportions of COD monomer and G2 loadings for fabricating printable co‐prepolymer inks. f) Relative viscosity and shear‐thinning properties of the co‐prepolymer inks.

Tuning the ink rheological properties to match or exceed the matrix viscosity is essential for successful EMB3D.^[^ ^47^, ^48^ ^]^ Traditional methods rely on the incorporation of rheology modifiers, curing agents, reactive diluents, or other additives, which inevitably affect the properties of the printed material. To circumvent these issues, rheology modification without altering the intrinsic material properties is necessary. **Figure** **4** illustrates that partial polymerization of the DCPD or COD inks results in optimal fluid properties for EMB3D in the chemical curing matrix. Rather than incorporating additives such as FS to convert the ink into a yield stress fluid, controlled pre‐polymerization generates a viscous liquid capable of shape retention after exposure to an activating matrix solution. We observed how printed filament morphology changes depending on whether the ink's apparent viscosity is much lower or similar to that of the matrix (Video S2, Supporting Information). Without rheology modification for both ink and matrix, the printed filament breaks into droplets due to the surface tension. When unmodified ink is printed in a matrix with yield stress, a fin‐shaped morphology is observed, characterized by a sharp edge at the top and a broader base at the bottom as the ink flows behind the nozzle. However, when the ink's zero‐shear viscosity is tuned through partial polymerization to approximate the high shear viscosity of the matrix, the filament retains a cylindrical shape and maintains structural integrity for the time required for the skin formation, without the need for additives or rheology modifiers.

In traditional batch polymerization of DCPD or COD, 0.8 mm of second‐generation Grubbs catalyst ([(SIPr)Ru(= CHPh)(PCy_3_)Cl_2_]; G2) is required for full monomer conversion. At significantly lower G2 concentrations (< 5.6 µm), however, only partial polymerization occurs with a corresponding increase in the resin viscosity (Figure 4a). By adjusting the G2 concentration, the zero‐shear viscosity of the DCPD or COD monomer solutions can be tuned over three orders of magnitude. For DCPD, G2 concentrations range from 2.4 to 4.8 µm, while for COD, they range from 50.4 to 74.4 µm to achieve similar viscosities (Figure 4b). COD ink requires ≈20 times the G2 loading of DCPD ink to achieve similar viscosity because COD predominantly generates linear strands. In contrast, DCPD can form branched structures after ring‐opening metathesis of pendent cyclopentene fragments, which increase viscosity more significantly than purely linear analogs.

Size exclusion chromatography (SEC) analysis demonstrated that while the ink primarily consists of monomers, the proportion of polymers increases with the rising concentration of the G2 (Figure S11, Supporting Information). The molecular weights (M_n_) of the polymers are ≈  5 × 10^5^ g mol^−1^ for DCPD ink and ≈ 2 × 10^5^ g mol^−1^ for COD ink. Both viscosity‐tuned inks exhibit shear‐thinning behavior (Figure 4c; Figure S12, Supporting Information). The increase in viscosity during the reaction was measured at different G2 loadings, showing that it reaches a plateau after 10 min of reaction time at 80 °C (Figure S13, Supporting Information). The storage modulus (G′) of the resulting viscous resins, with G2 concentrations >5.6 µm, exceeds the loss modulus (G′′), indicating that the resin reaches the gel point even at these low G2 concentrations.

The exothermic heat of polymerization, as measured by differential scanning calorimetry (DSC), is proportional to the initial G2 loading (Figure S14, Supporting Information). The initial G2 concentration dictates the resin's viscosity and maintains it at that level, with minimal further reaction and negligible viscosity change over several months (Figure S15, Supporting Information). Importantly, the viscous resins can still fully polymerize upon the addition of the remaining G2 catalyst (≈ 0.8 mm), indicating that most of the monomers retain their ability to undergo exothermic reactions for full conversion (Figure S16, Supporting Information). The size distribution of the prepolymer, measured by dynamic light scattering (DLS), ranged from 43 to 107 nm (Figure S17, Supporting Information). Additionally, the amount of polymer precipitated increased beyond a G2 loading of 2.4 µm (Figure S18, Supporting Information). Fourier‐transform infrared spectroscopy (FTIR) and NMR spectroscopy analyses of the prepolymer resins were employed to confirm the chemical structure and composition of the prepolymer resins (Figures S19 and S20, Supporting Information).

The same strategy was applied to prepare DCPD/COD co‐prepolymer inks for printing polymers with tunable mechanical properties.^[^ ^49^ ^]^ DCPD and COD monomer mixtures in various ratios were partially polymerized to create co‐prepolymer inks with desirable viscosities (Figure 4d). The resulting co‐prepolymers exhibited varying DCPD and COD compositions, forming either DCPD‐rich or COD‐rich inks as formulated (Figure S21, Supporting Information). As the fraction of COD increased (Figure 4e), a higher G2 concentration was required to achieve the desired viscosity for printing (≈100 Pa s). Interestingly, the DCPD/COD comonomer solution exhibited a linear relationship between the COD fraction and the G2 loading required to achieve similar viscosity, except for the pure COD monomer solution, which required 66 µm G2 loading—1.5 times higher than predicted by the linear relationship. This deviation is attributed to DCPD's significantly greater contribution to viscosity due to its branching at the cyclopentene ring. SEC of DCPD/COD inks revealed that the M_n_ decreases from 5 × 10^5^ g mol^−1^ to 2 × 10^5^ g mol^−1^ while the polymer fraction increases with rising concentrations of COD and G2 (Figure S22, Supporting Information). This supports earlier findings that the viscosity of DCPD inks is significantly affected even at lower G2 concentrations. The observation aligns with the previous results on DCPD inks, suggesting that the branching in DCPD substantially influences viscosity even at lower G2 concentrations. The resulting inks exhibited shear‐thinning behavior, with the COD‐rich resin requiring a higher shear rate to flow (Figure 4f). The relative viscosity (η/η_0_) varied with shear rate across different COD ratios, indicating that more branched polymers exhibited more pronounced shear thinning compared to less branched polymers. The presence of hyperbranched polymers reduced the critical shear rate for shear thinning, suggesting fewer entanglements within the system.^[^ ^50^, ^51^, ^52^ ^]^

### 2.5 Fabrication of Thermosets with Defined Properties

**Figure 5:**
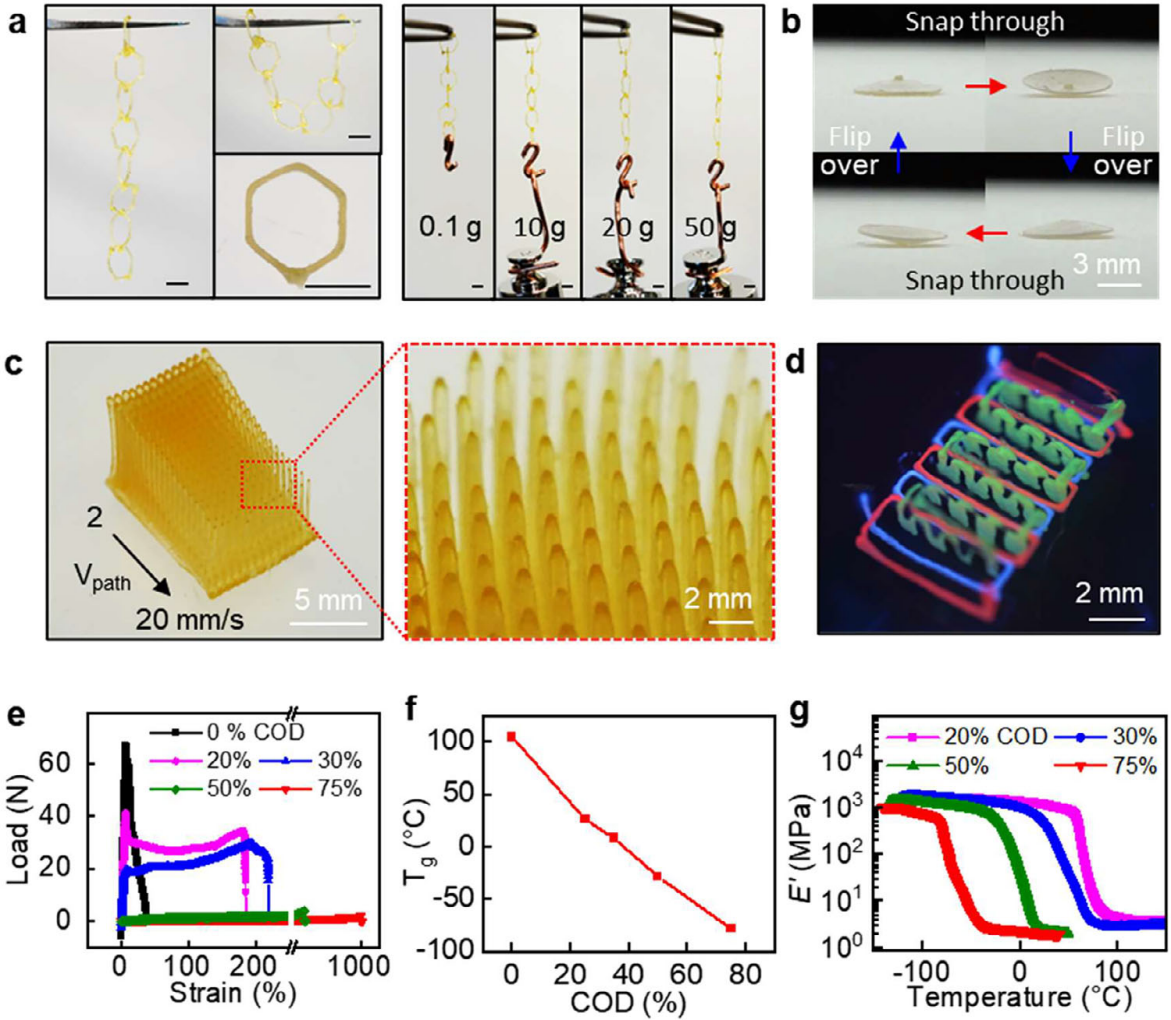
Printed and cured copolymers and their properties. a) Chain structures demonstrating the weight‐bearing capacity of DCPD‐rich formulations (scale bars 2 mm). b,c) Printed complex geometries including (b) shallow spherical shell structure exhibiting snap‐through buckling behavior and c) hair‐like structures at different printing speeds. d) Multi‐material printing within a single matrix solution (under UV light). e) Stress–strain curves of p(DCPD‐*co*‐COD). f) Glass transition temperatures and g) thermomechanical properties of copolymers with varying COD ratios.

**Figure** **5** illustrates polymers with varying properties that were printed, cured, and subsequently removed from the chemical curing matrix. The ability to print intricate structures with fine resolution and integration of multiple materials within a single curing bath highlights the versatility and precision of this method. It can also be used to fabricate interpenetrating features, enabling the creation of unconnected yet interlinked structures. For example, a chain structure composed of eight hexagonal rings made from DCPD ink demonstrates flexible, interlinked components that remain unconnected but structurally supported by the surrounding bath (Figure 5a). Despite its low mass (4 mg), the structure exhibits robust mechanical properties, capable of supporting a load 12 000 times its own weight (50 g). Notably, this approach eliminates the need for auxiliary support structures and minimizes printing waste to print movable parts.

Figure 5b depicts a shallow spherical shell structure fabricated via EMB3D using DCPD ink. When a compressive force is applied to the center, the structure exhibits snap‐through buckling behavior, enabled by the material's flexibility and toughness, and the shell geometry. This response is particularly notable, as the printed structure maintains mechanical integrity and bi‐stability despite having minimal thickness (200 µm). The shell undergoes a smooth and reversible snap‐through transition under applied load, which can be repeated multiple times, highlighting the structure's reversible bi‐stability (Video S3, Supporting Information). The shallow curvature of the shell shows the precise control during printing and limited deformation during curing.

The spatial resolution of the EMB3D approach is primarily governed by the interfacial tension (*Γ*) and yield stress of the supporting matrix (*σ_y_*), which determine the resistance to filament deformation and collapse. These parameters define the plastocapillary number *Y*_Γc_ = σ_*y*_ *d*_min_/2Γ , which sets the theoretical lower limit of printable feature size.^[^ ^53^ ^]^ However, due to rapid interfacial solidification of the ink, we achieved smaller filament diameters than predicted, down to ≈5 µm by employing nozzles with smaller inner diameters (Figure S23, Supporting Information). Recent work by Eom *et al.* ^[^ ^54^ ^]^ demonstrated similar strategies using solvent exchange to achieve features down to 1.5 µm, suggesting that future reduction is feasible with higher‐viscosity inks and faster curing kinetics. Future efforts could reduce minimum feature sizes below ≈5 µm by accelerating activator diffusion or designing faster‐reacting matrix chemistries.

Figure 5c illustrates the fabrication of hair‐like structures that mimic ear hair cells, demonstrating the ability to print free‐standing, high aspect ratio geometries without requiring woven or mesh support structures.^[^ ^54^, ^55^ ^]^ The nozzle speed was adjusted from 2 to 20 mm s^−1^, allowing for the control of hair diameters from 400 to 100 µm, which corresponds to aspect ratios between 25 and 100. The spacing between fins was fixed at 0.5 mm, and the height of each structure was set to 5 mm. The filament diameter can be further reduced to a few micrometers by employing nozzles with smaller inner diameters (Figure S23, Supporting Information). This was enabled by the use of higher zero‐shear viscosity DCPD inks, which increase the capillary breakup timescale relative to the diffusion timescale.^[^ ^53^, ^54^ ^]^ The printing parameters were optimized by adjusting the ink flow rate and the translational speed of the nozzle head. Typically, when the path velocity (V_path_) does not align with the output velocity (V_out_), instabilities arise.^[^ ^56^ ^]^ If the printing speed exceeds the material supply rate, it can cause the filament to stretch and fragment into droplets, resulting in discontinuous printing. Conversely, if the printing speed is too slow, it leads to overdeposition, resulting in filaments with rough surfaces, irregular morphologies, and inconsistent diameters. Remarkably, as shown in Figure S24 (Supporting Information), the shape of the printed filaments in the chemical curing matrix remained cylindrical, consistently with minimal distortion or winkling,^[^ ^54^ ^]^ whereas the diameter increases with decreasing print speeds across a range of 1 to 15 mm s^−1^. The extruded ink remains flowable and can adjust its shape due to cohesive forces after deposition, before forming a crust layer to maintain the cylindrical shape.

Furthermore, the technique enables multi‐ink printing within the same matrix, as demonstrated in Figure 5d. This capability allows for the sequential addition of different materials without significantly disturbing the pre‐existing structures. Printing multiple materials in a single curing matrix offers significant advantages for creating composite structures with varying mechanical properties and functionalities. To evaluate the compatibility of the EMB3D process with functional composite systems, we incorporated conductive fillers such as carbon nanotubes (CNTs) or silver nanowires (AgNWs) into the ink formulation. These composites (1 and 20 wt% loadings, respectively) cured successfully under standard conditions, indicating that the presence of conductive additives did not inhibit interfacial polymerization. The CNT fillers remained uniformly distributed when a high activator concentration was used (11 mm). Interestingly, under slow curing conditions (1.1 mm activator concentration), the fillers exhibited inward migration toward the center as the polymerization front progressed from the interface (Figure S25, Supporting Information). This behavior was further explored by stacking filaments into planar geometries, where AgNW filler concentration increased at the midplane, forming a percolated network (Figure S26, Supporting Information). Such spatial segregation can be advantageous, as it enables enhanced electrical conductivity with lower filler content while retaining the insulating character of the surrounding polymer matrix. Additionally, by depositing the composite ink on top of the bath rather than fully embedding it, the AgNWs preferentially accumulated at the upper surface, away from the activator‐rich interface (Figure S27, Supporting Information). This produced a conductive top layer with sheet resistance as low as ≈4 Ω sq^−1^, highlighting the potential of this approach for structured conductive patterning.

As a demonstration of EMB3D of elastomers, co‐prepolymer inks were utilized to fabricate elastomers with varying mechanical properties and *T_g_*. The p(DCPD‐*co*‐COD) materials with differing COD content were fabricated by utilizing co‐prepolymer inks that contain 0, 20, 30, 50, and 70 wt% COD, and were assessed through tensile testing (see Experimental Section). Although the geometries between samples are similar (≈20 mm × 7 mm × 0.3 mm), measuring cross‐sectional areas accurately is challenging due to the inherent 3D printing process that stacks cylindrical filaments, complicating the determination of tensile modulus values. Cross‐sectional images of the printed films reveal continuous and well‐integrated structures between adjacent filaments (Figure S28, Supporting Information), revealing no visible interlayer interfaces, indicating effective interfacial bonding after curing. While this limits the accurate evaluation of tensile modulus, the tensile load and elongation at failure exhibit clear dependence on the COD content in the ink formulation (Figure 5e). Copolymer samples with 0, 20, 30, 50, and 70 wt% COD failed at loads of 67.2, 41.0, 29.5, 3.7, and 1.7 N, respectively, with elongations at failure of 37%, 185%, 218%, 413%, and 1011%, respectively. The 0 wt.% COD sample, p(DCPD), did not exhibit cold drawing behavior as previously reported,^[^ ^49^ ^]^ likely due to the use of a different catalyst and the absence of phenylcyclohexane, which in previous studies acted as a solvent to solubilize the catalyst and may have contributed to network plasticization. Notably, structures fabricated from ink with 75 wt% COD demonstrated the capability to stretch to 1000% of their original shape. Copolymers with a COD fraction below 25 wt% demonstrated cold‐drawing behavior, whereas those above 30 wt% showed elastomeric behavior, similar to that previously observed in DCPD‐based thermosets prepared by FROMP.^[^ ^57^ ^]^ However, poly(1,4‐butadiene) derived from 100% COD was excluded from the study due to irrecoverable deformation, attributed to the macroscopic flow of polymer chains.^[^ ^58^ ^]^ The glass transition temperature (*T_g_*) values of the copolymers exhibited a broad range between −78 and 105 °C (Figure 5f), as determined by DSC. The *T_g_* variation with comonomer composition closely adhered to the Fox equation for random copolymers (Equation S1, Supporting Information), indicating that the two comonomers were statistically incorporated into the copolymer without phase separation during reaction. The consistent variation in *T_g_* with comonomer ratio highlights the precise control over thermal properties achievable through the EMB3D in a chemical curing matrix and copolymerization technique. Thermomechanical properties measured through dynamic mechanical analysis (DMA) also confirmed that *T_g_* can be adjusted by varying the COD fraction in the polymer (Figure 5g). This level of control stems from the tunable crosslink density enabled by adjusting the DCPD/COD comonomer ratio, which governs the resulting mechanical properties. Because this parameter is independent of printing conditions, it allows spatially programmed material behavior without compromising printing fidelity. Such programmable tunability enables the design of heterogeneous structures with integrated stiff and compliant regions, beneficial for applications like soft robotics, wearable devices, and architected materials that require localized mechanical gradients.

### 2.6 EMB3D Limitations and Opportunities

While EMB3D enables the fabrication of complex thermoset architectures, several considerations define its operational boundaries. The spatial resolution and layer thickness are constrained by the diffusion length of the activator. As such, filaments thicker than ≈400 µm may not fully cure due to limited activator transport. To ensure uniform polymerization, multilayer and multimaterial structures were printed within this diffusion‐limited regime. Enclosed geometries, such as sealed cavities, pose challenges as matrix removal post‐curing becomes impractical without compromising structural integrity. Furthermore, while lines can be printed quickly, the small line diameter limits the rate at which material can be printed.

Enhanced activator mass transport or faster interfacial reaction rates would enable thicker lines. Secondary post‐curing strategies, e.g., thermal activation, microwave treatment, or photo‐triggered curing of latent additives in the ink, could complement the primary curing process and enable bulk solidification. Further formulation improvements could reduce catalyst loading (now ≈100 ppm Ru), explore alternative ROMP‐active metals such as W, Mo, or Fe,^[^ ^59^, ^60^, ^61^, ^62^ ^]^ and increase sustainability through bath reuse. Hardware‐level strategies such as multi‐nozzle printing systems e.g., as demonstrated by Lewis et al.^[^ ^22^, ^63^ ^]^ could significantly enhance throughput by parallelizing deposition.

Beyond these technical considerations, EMB3D offers distinct advantages for fabricating multi‐material thermoset structures with tunable mechanical properties we have only started to explore. EMB3D supports integration of both rigid and compliant domains spanning glassy to rubbery moduli at room temperature. This capability is particularly relevant for soft robots that require both stiff supports and flexible joints, wearable devices with embedded stretchable regions, and bioinspired systems that mimic the hierarchical mechanics of natural tissues. The method's compatibility with functional fillers further expands its scope to stimuli‐responsive and conductive composites, enabling applications in soft actuators, architected metamaterials, biomedical scaffolds, and biomimetic sensors such as artificial hairy interfaces.

## 3 Conclusion

In conclusion, we successfully demonstrated EMB3D using a chemical curing matrix and latent catalyst ink. This diffusion‐activation strategy circumvents many limitations of existing 3D printing methods of soft thermosets. Engineering the formulation and properly selecting the matrix solution facilitates the formation of a good interface while allowing for the diffusion of activator molecules through the interface. The activator effectively cures the printed filament with a diameter of <400 µm when the formulation is in stoichiometric concentration, with curing speed being proportional to the catalyst and activator concentration. The ink formulation was varied by adjusting the ratio of DCPD to COD, and viscosity was tuned through partial co‐polymerization. The printed and cured copolymer exhibited varied mechanical properties and *T_g_*, allowing for both rigid and soft mechanical properties. Intricate structures, such as chain structures or hairy structures, as well as composite prints, were successfully printed with the system described here. Inspired by the selective diffusion mechanisms observed in biological systems, the techniques developed in this research could be further expanded to include diverse materials and chemistries, along with a comprehensive range of biologically relevant materials.

## 4 Experimental Section

Experimental details are provided in the Supporting Information.

## Conflict of Interest

The authors declare no conflict of interest.

## Supporting information



Supporting Information

Supplemental Video 1

Supplemental Video 2

Supplemental Video 3

## Data Availability

The data that support the findings of this study are available in the supplementary material of this article.
