# Supplementary material for: Multimaterial 3D Printing in Activating Bath Enables In Situ Polymerization of Thermosets with Intricate Geometries and Diverse Elastic Behaviors
Source: Adv Mater. 2025 Aug 13;37(43):e08568. doi: 10.1002/adma.202508568 (PMC12574649; doi:10.1002/adma.202508568)
Supplement: Supplementary file 1 — Supporting Information [file ADMA-37-e08568-s003.pdf]

ADVANCED MATERIALS

Supporting Information

for *Adv. Mater.*, DOI 10.1002/adma.202508568

Multimaterial 3D Printing in Activating Bath Enables In Situ Polymerization of Thermosets with Intricate Geometries and Diverse Elastic Behaviors

*Young Bum Lee, Yun Seong Kim, Chen Chen, Mohammad Tanver Hossain, Benjamin A. Suslick, Randy H. Ewoldt, Sameh H. Tawfick, Jeffrey S. Moore, Nancy R. Sottos and Paul V. Braun**

Supporting Information

Multimaterial 3D Printing in Activating Bath Enables In–Situ Polymerization of Thermosets with Intricate Geometries and Diverse Elastic Behaviors

Young Bum Lee[a,b,c,e], Yun Seong Kim[a,d,e], Chen Chen[a,c,e], Mohammad Tanver Hossain[a,d,e], Benjamin A. Suslick[a,f], Randy H. Ewoldt[a,d,e], Sameh H. Tawfick[a,d,e], Jeffrey S. Moore[a,b,c,e,f], Nancy R. Sottos[a,b,c,d,e], Paul V. Braun[a,b,c,d,e,f*]

[a] Beckman Institute for Advanced Science and Technology, University of Illinois Urbana–Champaign, Urbana, IL 61801, USA.

[b] Materials Research Laboratory, University of Illinois Urbana–Champaign, Urbana, IL 61801, USA.

[c] Department of Materials Science and Engineering, University of Illinois Urbana–Champaign, Urbana, IL 61801, USA.

[d] Department of Mechanical Science and Engineering, University of Illinois Urbana–Champaign, Urbana, IL 61801, USA.

[e] Grainger College of Engineering, University of Illinois Urbana–Champaign, Urbana, IL 61801, USA.

[f] Department of Chemistry, University of Illinois Urbana–Champaign, Urbana, IL 61801, USA.

Keywords: Embedded 3D printing, Thermoset, Elastomer, Chemical activation, Ring opening metathesis polymerization.

1. Experimental Section

Materials and chemicals

Dicyclopentadiene (DCPD), phenylcyclohexane (PCH), 1,5-cyclooctadiene (COD), 5-ethylidene-2-norbornene (ENB), 2nd generation Grubb's catalyst (G2, M204), $P(O^nBu)_3$, copper chloride ($CuCl_2$), ascorbic acid, ethylene glycol, diethylene glycol, triethylene glycol, polyethylene glycol (MW 200), propylene glycol, polypropylene glycol (MW 425 and 1000), isopropyl alcohol, paraffin oil, and 4,4'-methylenebis(2,6-di-tert-butylphenol) were purchased from Millipore Sigma and used without further purification steps. Rheology modifier (fumed silica, Aerosil R-805) was provided by Evonik Corporation. D899 catalyst was provided from Vipo S.A.S.

Prepolymer synthesis

DCPD was melted and liquidized by adding 5 wt% of ENB. The resulting solution was then filtered through a basic alumina column and degassed overnight. Before use, an antioxidant, 4,4'-methylenebis(2,6-di-tert-butylphenol), was added at a concentration of 0.5 wt% and dissolved into the solution. In a separate vial, G2 (0.08 mM) was dissolved in PCH at a rate of 500 μL per mg of G2 and stirred for 15 minutes. After this, an equivalent amount of TBP (0.319 μL per mg of G2) was added to the G2 solution. The G2 solution was then combined with the prepared monomer. For the pre-polymerization of DCPD, a catalyst loading of 2.4-4.8 μM (0.3-0.6% of 0.8 mM) was added and vortexed, followed by heating at 60 °C for 15 minutes. Similarly, for the DCPD prepolymer, heating at 35 °C for 60 minutes produces comparable results. For the pre-polymerization of COD, a catalyst loading of 50.4-74.4 μM (6.3% to 9.3% of 0.8 mM) was added and then heated at 60 °C for 15 minutes. Regarding comonomer inks, DCPD and COD solutions were mixed in various ratios (80/20, 70/30, 50/50, and 25/75 weight ratios). To these mixtures,

16.8, 20.8, 26.4, and 36 μM of catalyst solution (2.1% to 4.5% of 0.8 mM) were added, each followed by heating at 60 °C for 15 minutes.

Prepolymer ink preparation

Prepolymer solutions with the desired viscosity were transferred into separate vials. D899 catalyst at concentrations of 0.55, 1.1, or 3.3 mM and 1.5 equivalents of TBP were dissolved in these solutions. The prepared inks were then transferred into 3 cc barrels and maintained in a nitrogen environment prior to printing. For the embedded 3D printing process, 30 and 32 gauge printing nozzles (Nordson stainless tips) were used. Composite inks were prepared by blending 1 wt% MWCNT or 20 wt% of AgNWs into the prepolymer ink after solubilizing the D899 catalyst.

Chemical curing matrix preparation

0.55, 1.1, or 3.3 mM of copper chloride ($CuCl_2$) along with 1.5 equivalents of ascorbic acid were dissolved in the matrix solution. The ascorbic acid reduced the copper, changing the solution's color from blue to transparent. Various types of matrices such as EG, DEG, TEG, PEG, PG, and PO were tested for printing. To modify the rheological properties, 4.5 wt% of fumed silica (Aerosil R-805) was incorporated using a speedmixer (Hauschild SpeedMixer 150) at 2000 rpm.

Mutual solubility measurements

Nine matrix solutions listed in Table S1 were each mixed with an equal volume of a DCPD solution. The mixtures were allowed to equilibrate for 3 hours at room temperature to reach a steady state of solute distribution between the two phases. After equilibration, any small droplets or emulsions present at the interface were removed by centrifugation at 2000 rpm for 10 minutes

to ensure clear phase separation. For systems that exhibited phase separation between the matrix solution and the DCPD solution, aliquots were carefully taken from each phase using a pipette while minimizing cross-contamination. The collected samples were analyzed using Nuclear Magnetic Resonance (NMR) spectroscopy to quantify the concentration of minor components. Specifically, the amounts of DCPD dissolved in the matrix phase and vice versa.

Characterization

^{1}H NMR spectroscopy was used to analyze partitioning between printing ink and solvent. ^{1}H NMR samples were made by dissolving ~15 mg of the resin mixture in ≈ 0.7 mL of acetone-d_6 or chloroform-*d*. ^{1}H NMR spectra were collected on Carver B500 Bruker Advance III HD NMR Spectrometer equipped with a CryoProbe. Integrations of characteristic ^{1}H peaks were used to calculated molar ratio between species in mixture.

Rheological properties of prepolymer solutions and matrix solutions were measured in the TA Instruments DHR-3 rheometer equipped with a 25 mm flat plate and Peltier plate. Storage modulus (G') and loss modulus (G'') were measured with strain sweep analysis at 1 Hz. Yield stress was defined as the intersection point between storage modulus (G') and loss modulus (G'').

Molecular weights of the prepolymers were characterized by size exclusion chromatography (SEC) was performed on a Tosoh EcoSEC 8320 GPC system with tetrahydrofuran as the mobile phase. Fourier-transform infrared spectroscopy with attenuated total reflectance (FTIR-ATR) spectra were obtained using a Bruker Alpha Platinum ATR spectrometer. DSC was performed on a TA Instruments Discovery DSC 250.

To characterize in the printed polymers, thermogravimetric analysis (TGA) was performed using a TA Instruments Q50 analyzer. The temperature was increased from 22 °C to 600 °C at a

heating rate of 10 °C min^{-1}. Tensile dynamic mechanical analysis (DMA) was performed using a TA Instruments Q800 equipped with thin-film grips and a liquid nitrogen cooling system. The temperature was increased linearly from −90 °C to 150 °C at a rate of 2 °C min^{-1}. Dynamic loading was applied at a frequency of 1 Hz with a strain amplitude of 0.1%.

Embedded 3D printing

A 3D printing setup was employed utilizing an A3200 integrated automation motion system and G-code programming language. The printing process was controlled using an Ultimus V High Precision dispenser (Nordson EFD), which provided positive-pressure control, combined with 3cc amber light-blocking syringe barrels and 30-32 gauge stainless steel nozzles (Nordson). For high magnification imaging of the printed threads, an IDS USB 3.0 C-Mount Camera equipped with a color CMOS sensor, a 1.5x Navitar Attachment Lens, and a 2.0x Precise Eye Navitar Adaptor Lens (1stVision Inc.) was used. The printing area was illuminated by a 6 Watt LED Dual Gooseneck Illuminator (AmScope). Unless otherwise specified, a catalyst concentration of 3.3 mM and an activator concentration of 3.3 mM were used for printing.

2. Supporting Figures, Tables, and Discussion

Table S1. Mutual solubility values of nine matrix solutions (EG, DEG, TEG, PEG, PG, IPA, PPG425, PPG1000, and MO) and their capability for rheology modification utilizing fumed silica.

Sample number	Compound A	Compound B	Solubility of A in B $[A]_B$	Solubility of B in A $[B]_A$	Rheology modific ation	Print quality
1	DCPD/ENB	Ethylene glycol	0.63	0.03	Yes but turbid	Excellent
2	DCPD/ENB	Diethylene glycol	3.11	0.11	Yes	Unstable interface
3	DCPD/ENB	Triethylene glycol	4.25	0.27	Yes	Unstable interface
4	DCPD/ENB	Polyethylene glycol (MW 200)	5.71	0.64	Yes	Unstable interface
5	DCPD/ENB	1,2-propanediol	0.53	0.04	Yes	Excellent
6	DCPD/ENB	Isopropyl alcohol	Fully miscible		N/A	Poor
7	DCPD/ENB	Polypropylene glycol (MW 425)	Fully miscible		N/A	Poor
8	DCPD/ENB	Polypropylene glycol (MW 1000)	Fully miscible		N/A	Poor
9	DCPD/ENB	Mineral oil	Fully miscible		N/A	Poor

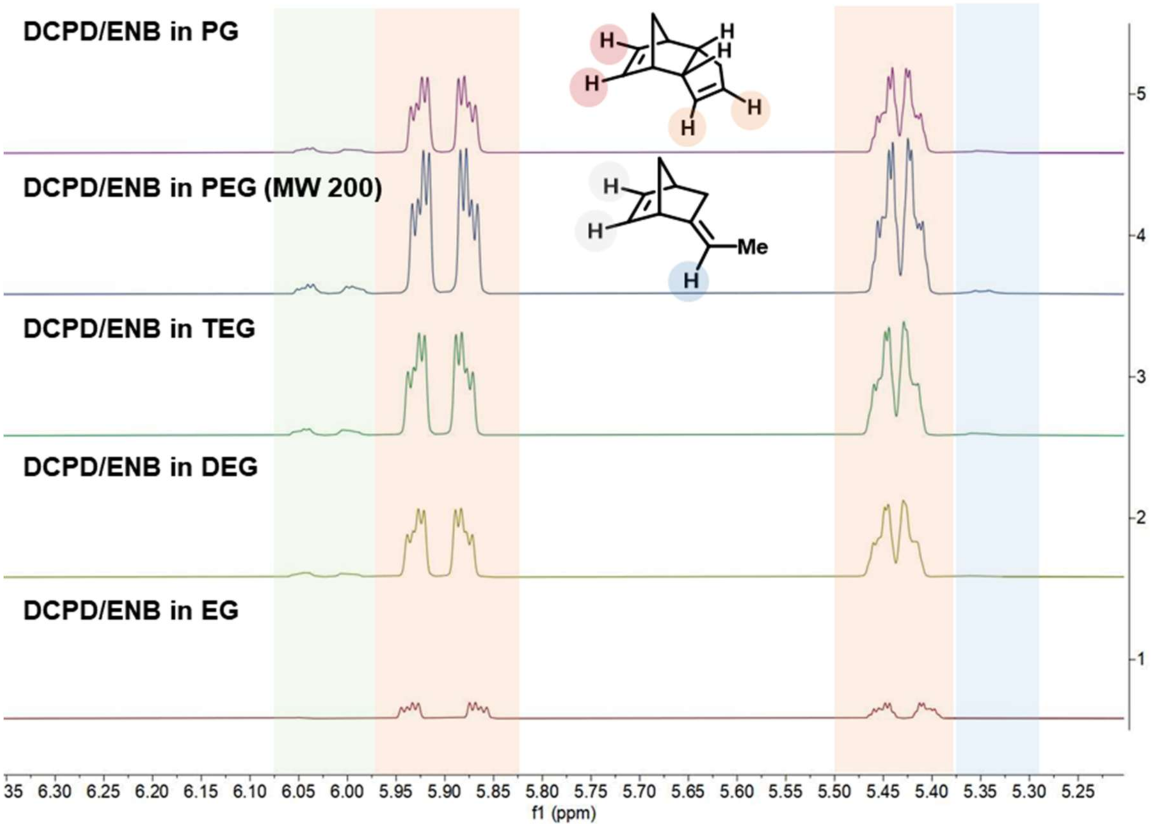

Figure S1. Stacked ^{1}H NMR spectra (acetone-d_6, 500 MHz) of matrix solutions after equilibration with DCPD solution. Characteristic peaks for DCPD and ENB present in PG, PEG, TEG, DEG, and EG solutions are shown in shaded regions.

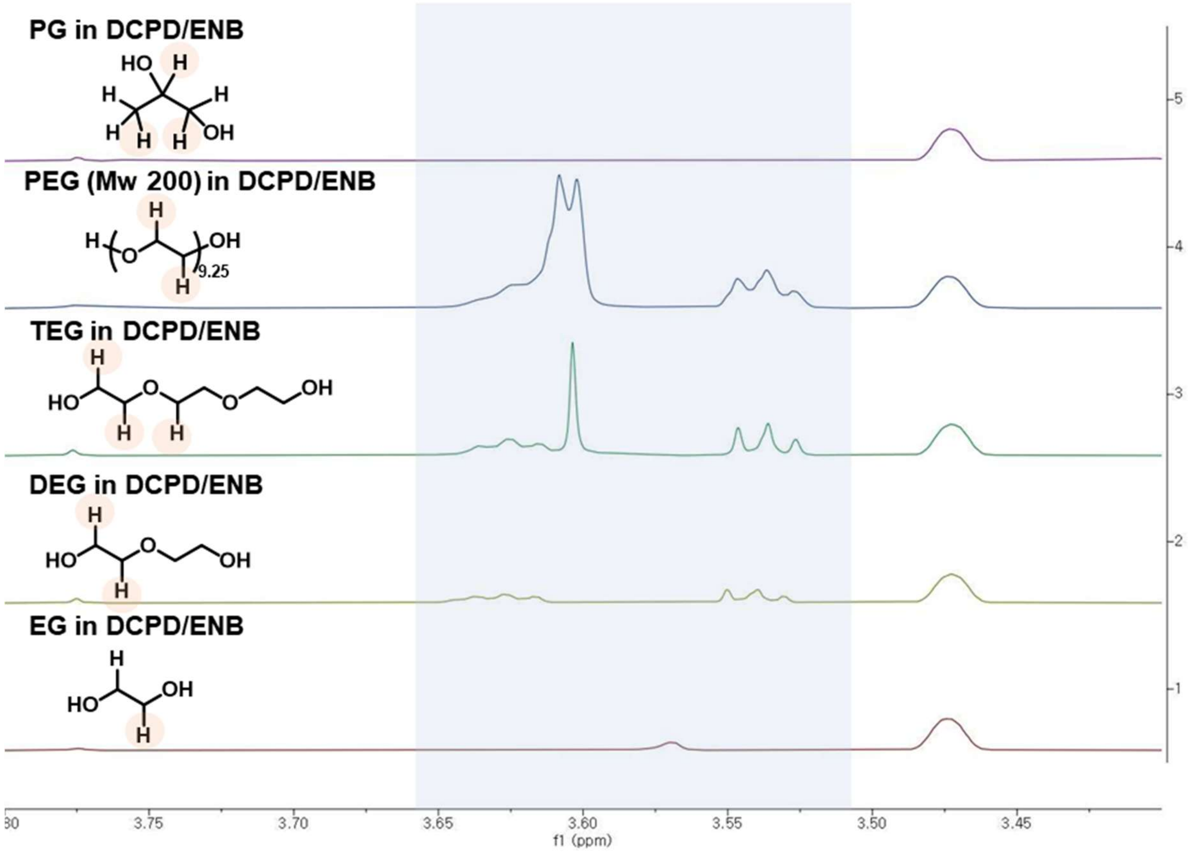

Figure S2. Stacked ^{1}H NMR spectra (acetone-d_6, 500 MHz) of DCPD solutions after equilibration with matrix solutions of PG, PEG, TEG, DEG, and EG. Characteristic peaks of matrix solutions present in DCPD are shaded.

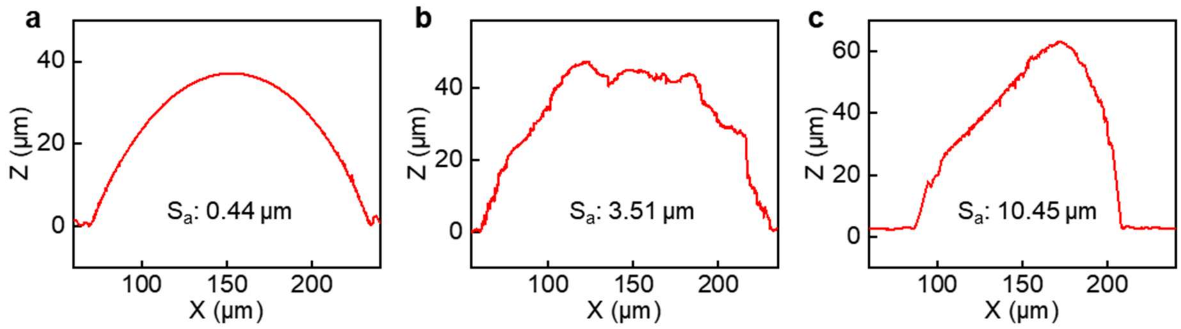

Figure S3. Line profiles and average surface roughness (S_a) of threads printed in matrix solutions of (a) PG, (b) PEG, and (c) MO matrix solutions, as measured by profilometry.

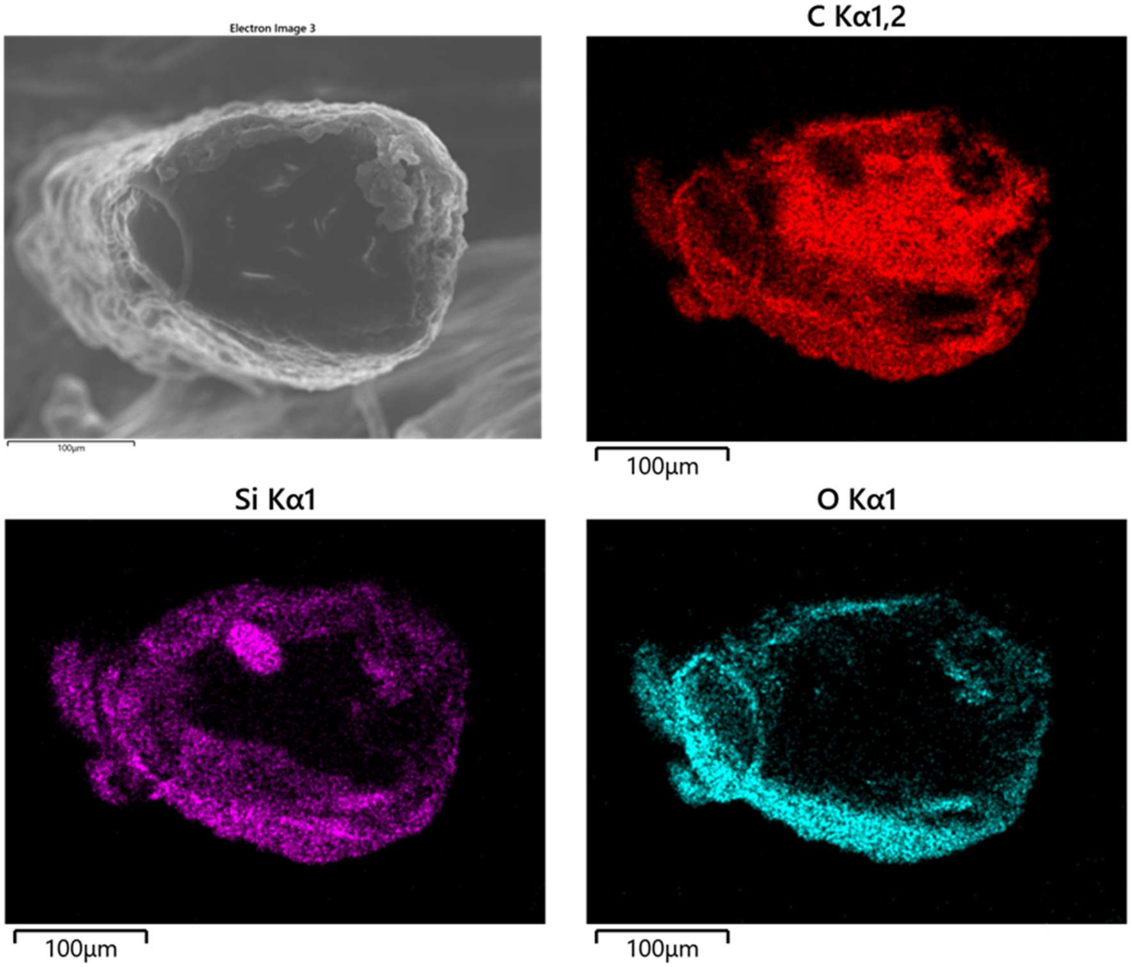

Figure S4. SEM cross-section and EDS mapping of a thread printed in PEG matrix.

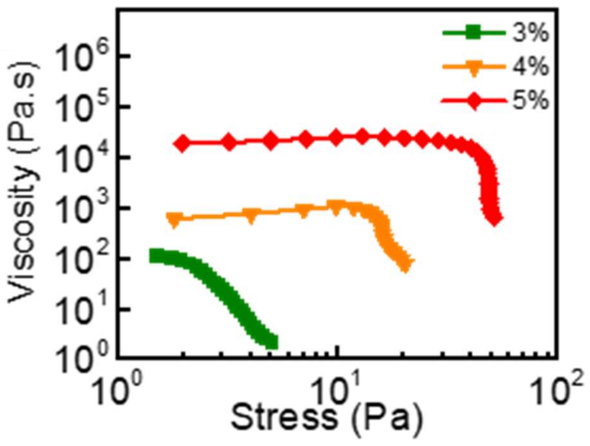

Figure S5. Large amplitude oscillatory shear (LAOS) test results, showing viscosity versus stress.

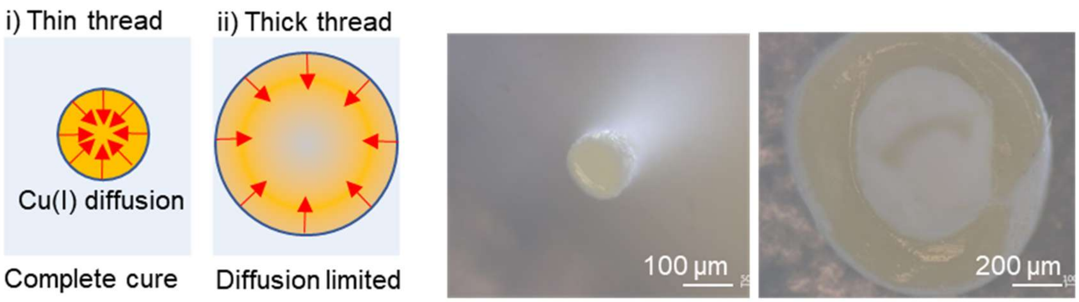

Figure S6. Cross-sectional images of printed features with diameters of 100 µm and 1 mm, taken after samples cured in the matrix followed by a subsequent washing with acetone.

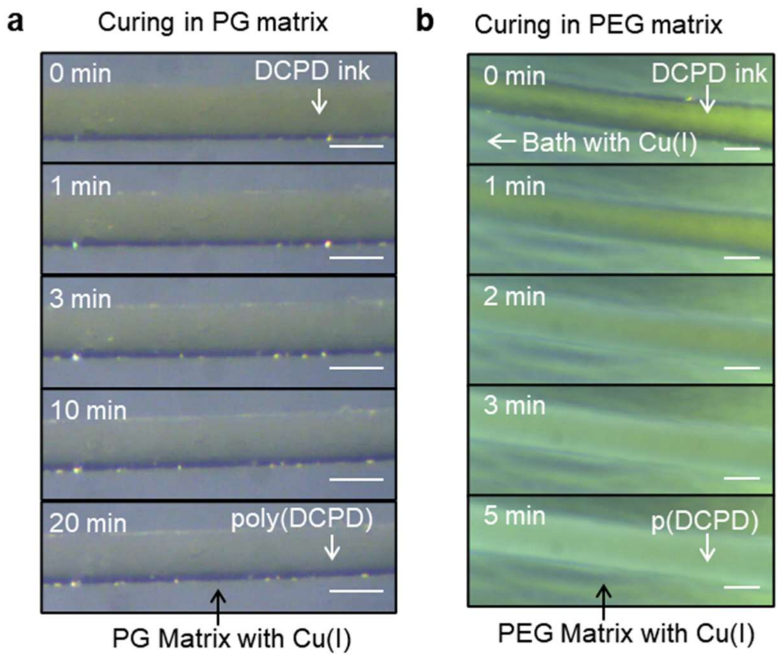

Figure S7. Snapshots of the curing process after deposition in (a) a PG matrix and (b) a PEG matrix (scale bars 50 μm).

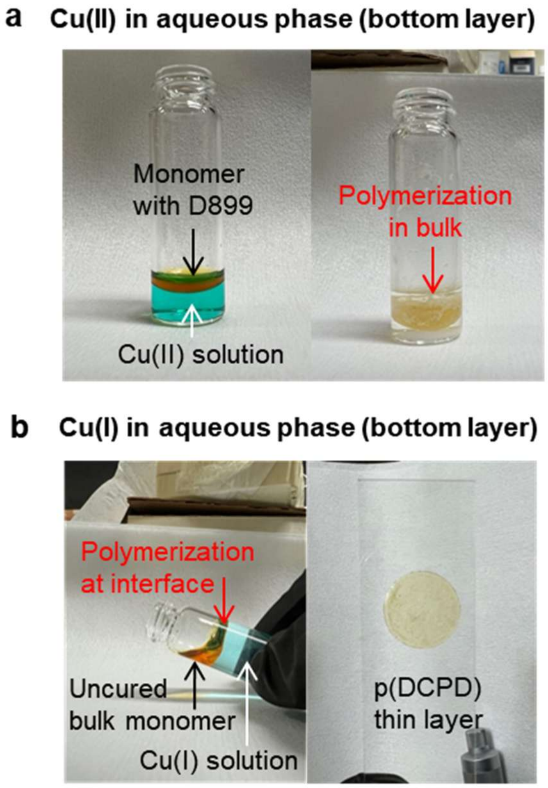

Figure S8. Interfacial polymerization between two phase-separated bulk solutions without rheology modifiers. The aqueous phase at the bottom contains (a) Cu(II) activator or (b) Cu(I) activator.

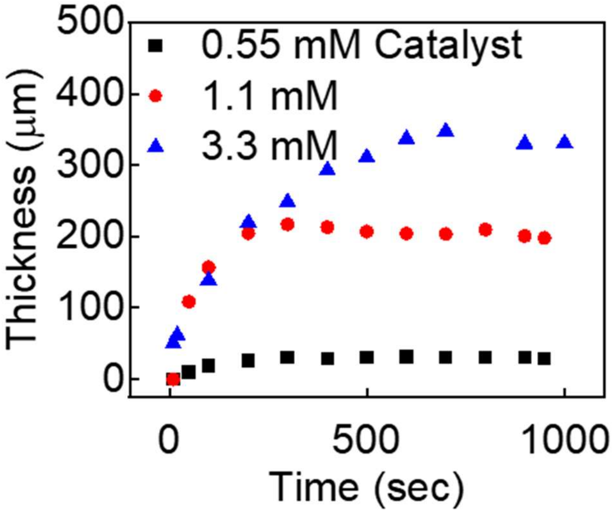

Figure S9. Measurements of film thickness over time for various catalyst concentrations (0.55, 1.1, and 3.3 mM) in a matrix containing 11 mM CuCl activator.

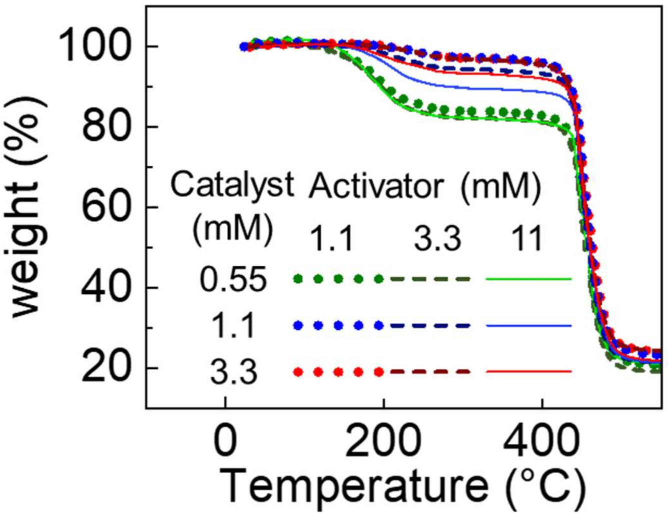

Figure S10. Thermogravimetric analysis (TGA) of samples containing catalyst concentrations of 0.55, 1.1, and 3.3 mM printed in matrices with activator (CuCl) concentrations of 0.55, 1.1, and 3.3 mM.

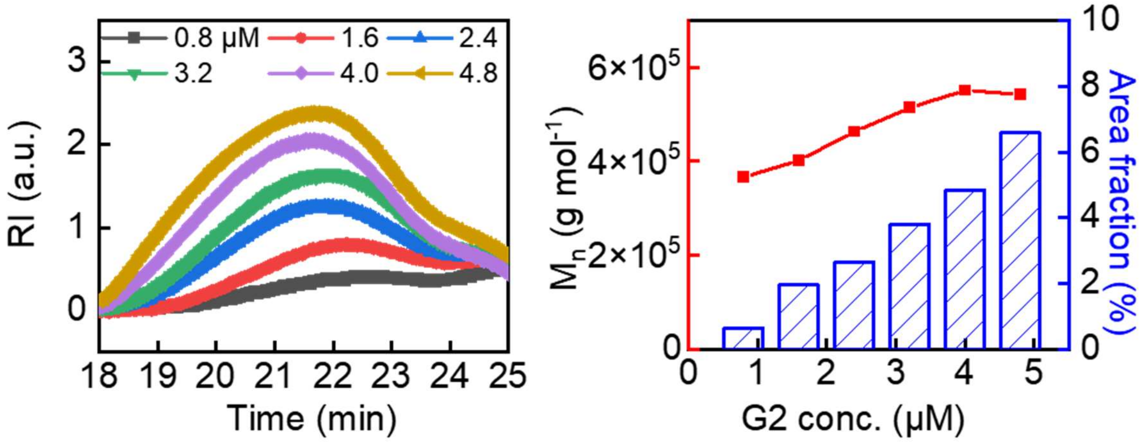

Figure S11. Size exclusion chromatography (SEC) analysis on DCPD inks.

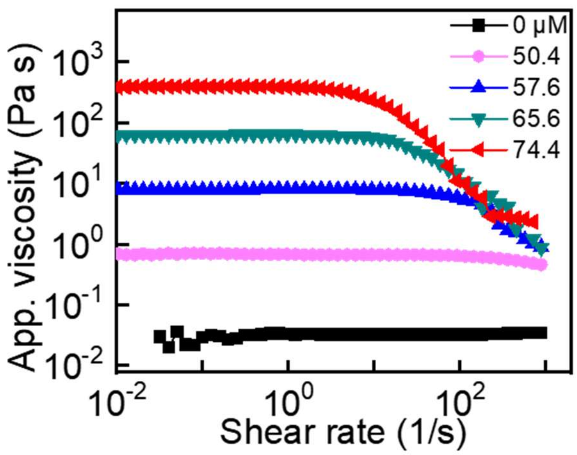

Figure S12. Rheological properties of COD inks after pre-polymerization with various G2 catalyst concentrations.

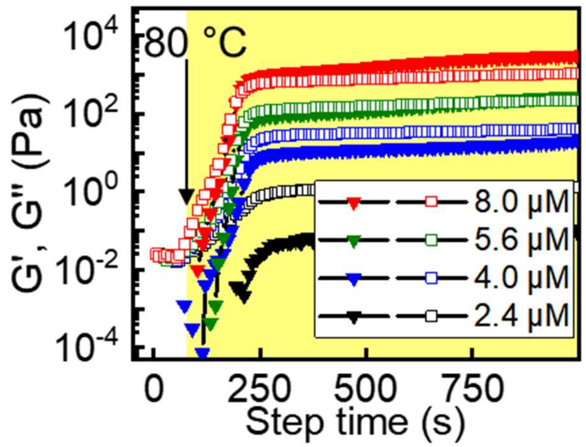

Figure S13. Evolution of viscosity during the reaction with various initial G2 catalyst loadings. Storage modulus (G', filled dots) and loss modulus (G'', empty dots) were monitored over 20 min.

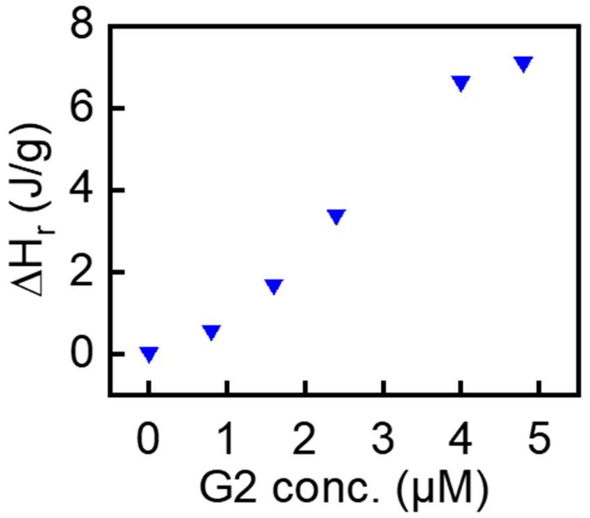

Figure S14. Heat of reaction (ΔH_r) during the prepolymerization of DCPD measured by DSC.

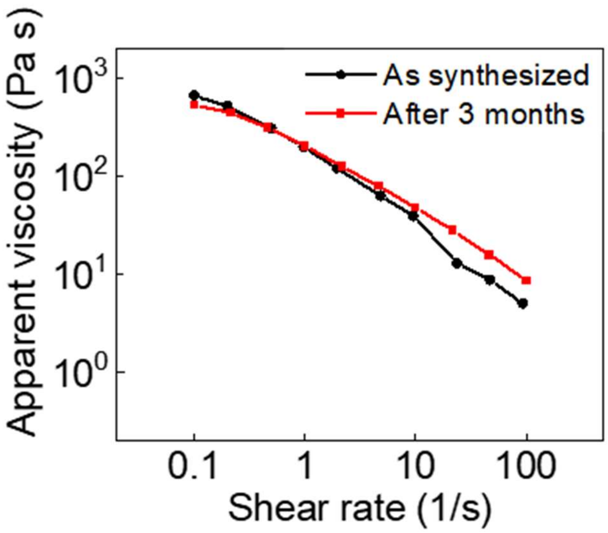

Figure S15. Rheological properties of inks (a) at synthesis and (b) after 3 months.

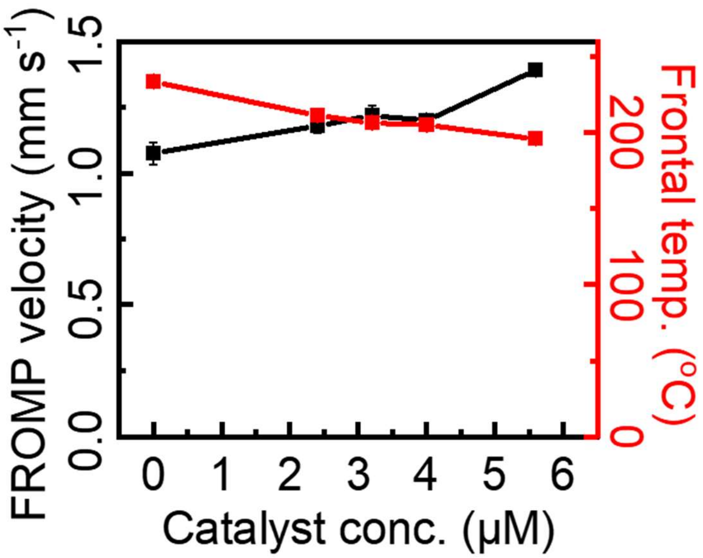

Figure S16. Frontal polymerization velocity and front temperature of the ink following the addition of 0.8 mM G2 catalyst.

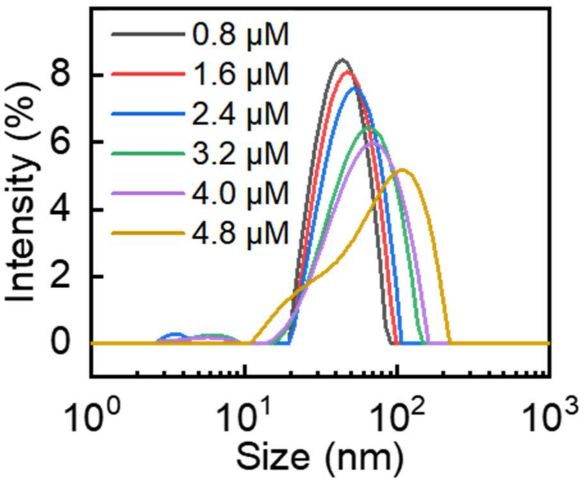

Figure S17. Size distribution of prepolymers measured by dynamic light scattering (DLS).

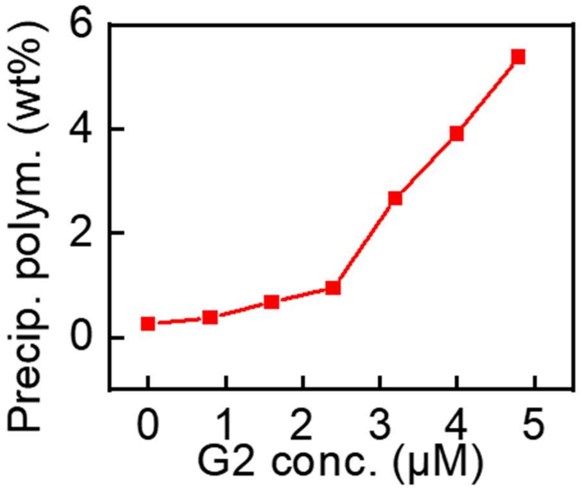

Figure S18. Amount of polymer precipitated from prepolymer inks with various initial catalyst concentrations after precipitation in ethyl ether.

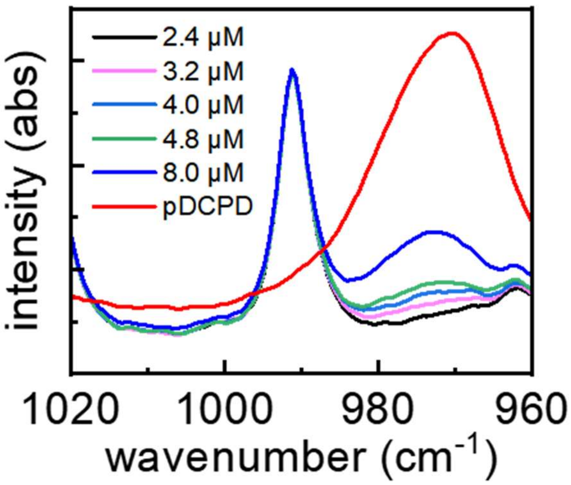

Figure S19. FTIR measurements on DCPD prepolymer inks and p(DCPD).

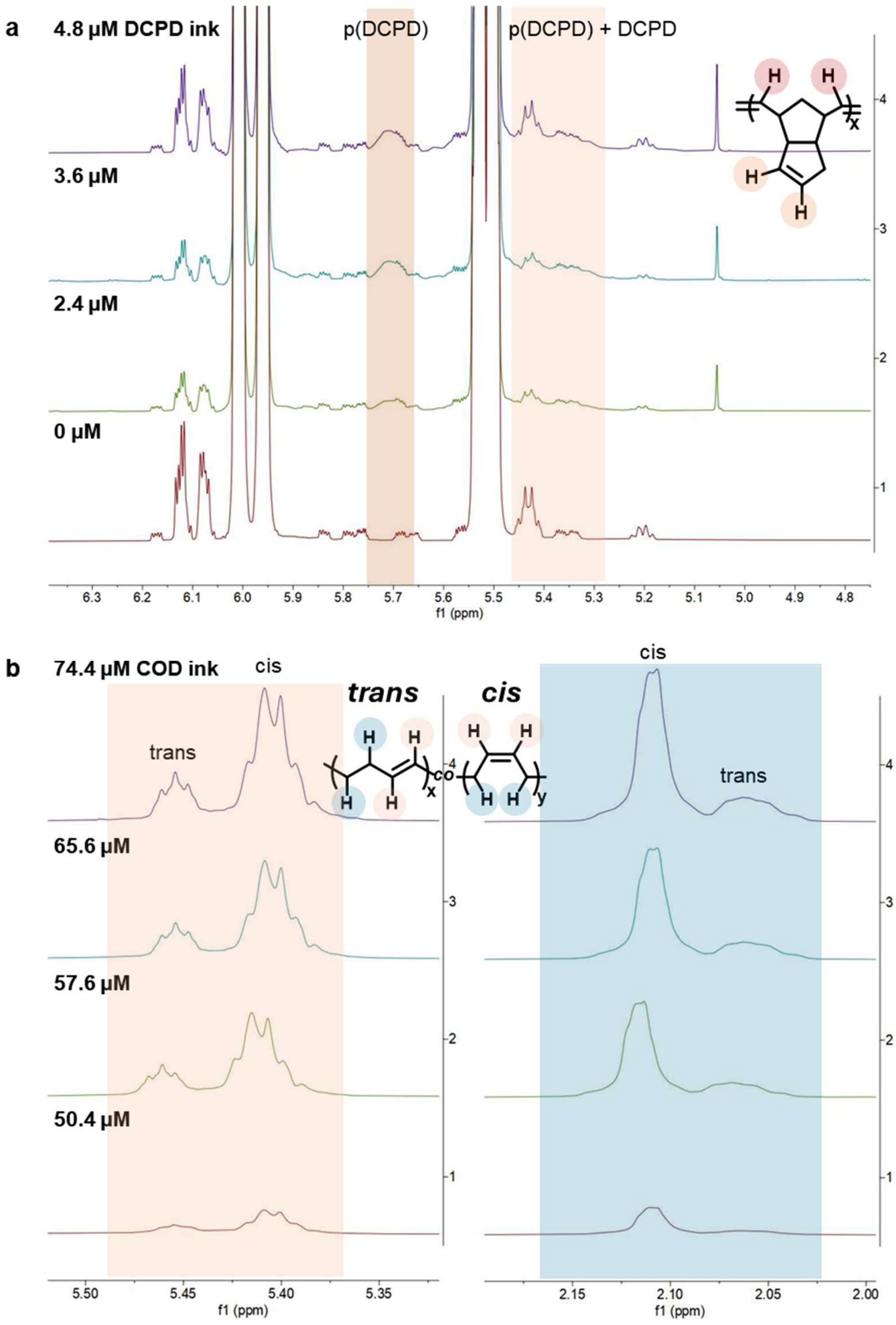

Figure S20. ^{1}H NMR spectra (chloroform-*d*, 500 MHz) of (a) DCPD prepolymer solutions (b) COD prepolymer solutions. *Cis*- and *trans*-conformations were determined by comparing the spectra with those from previous work.[1]

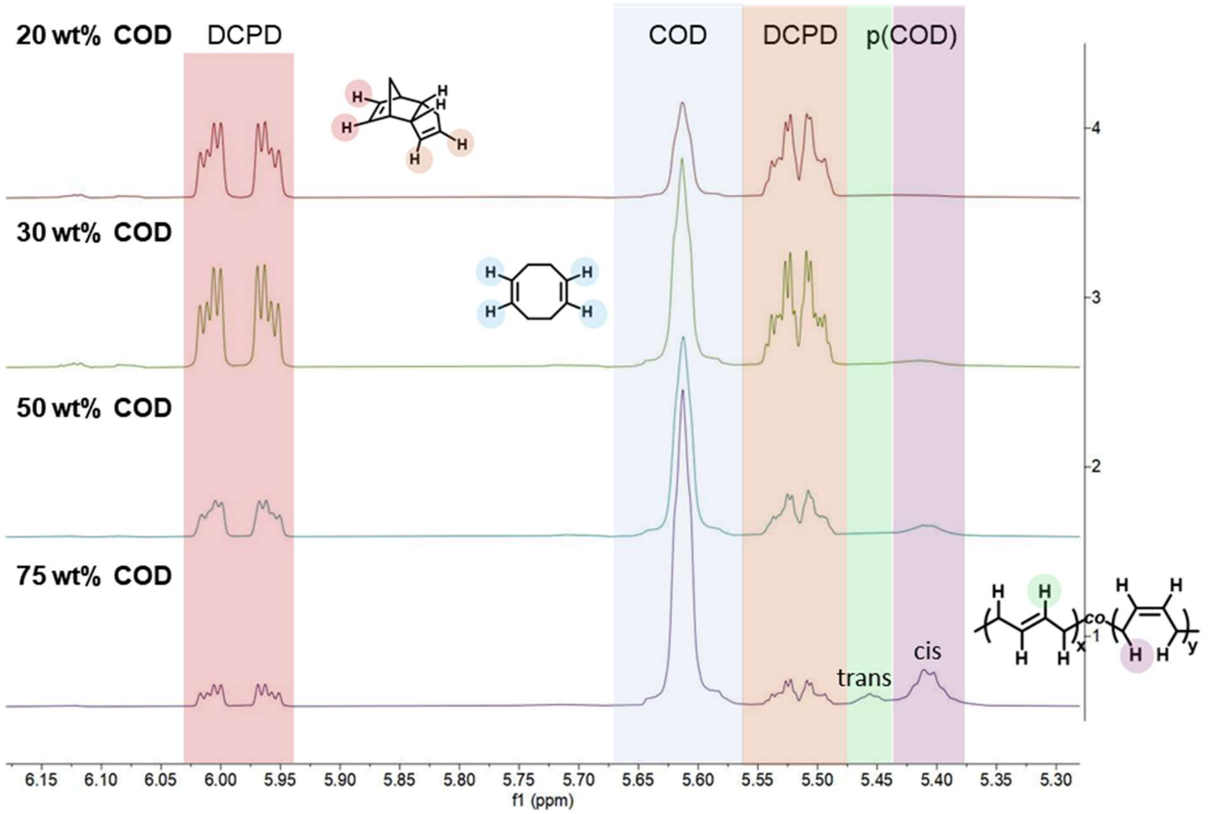

Figure S21. ^{1}H NMR spectra (chloroform-*d*, 500 MHz) of DCPD/COD co-prepolymer solutions containing 20, 30, 50, and 75 wt% COD.

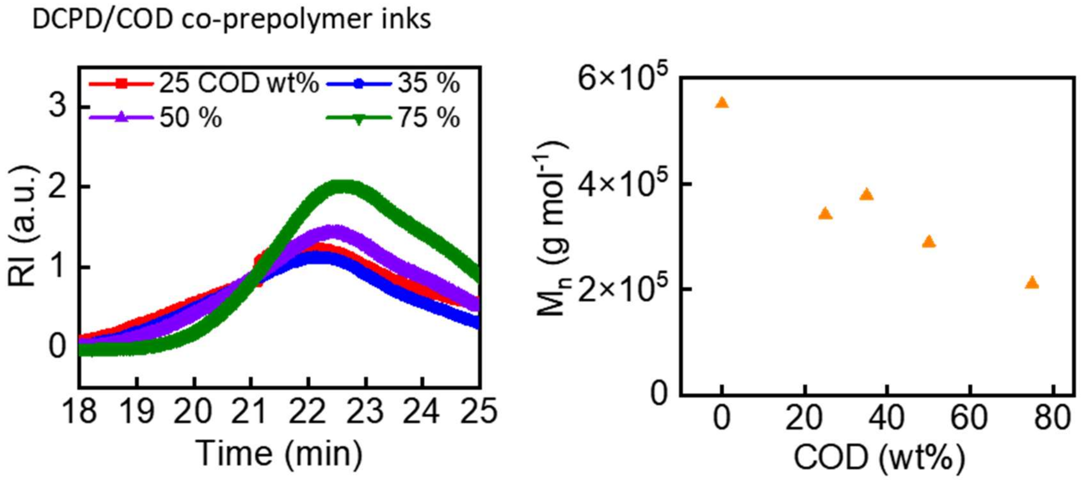

Figure S22. SEC analysis of co-prepolymer solutions with varying DCPD and COD compositions.

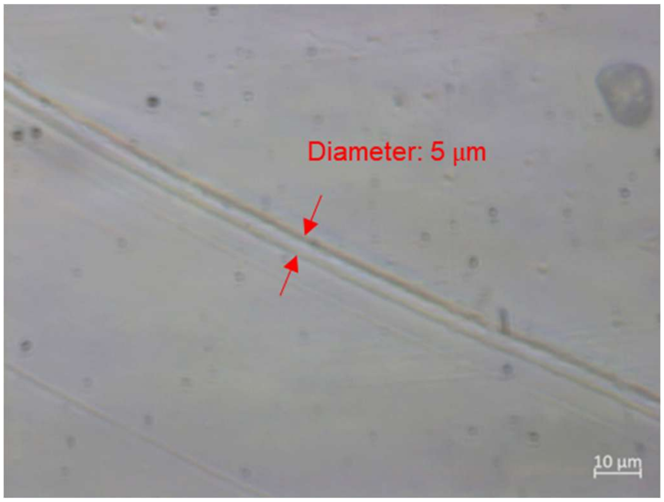

Figure S23. Printed polymer thread extruded using a 5 μm nozzle.

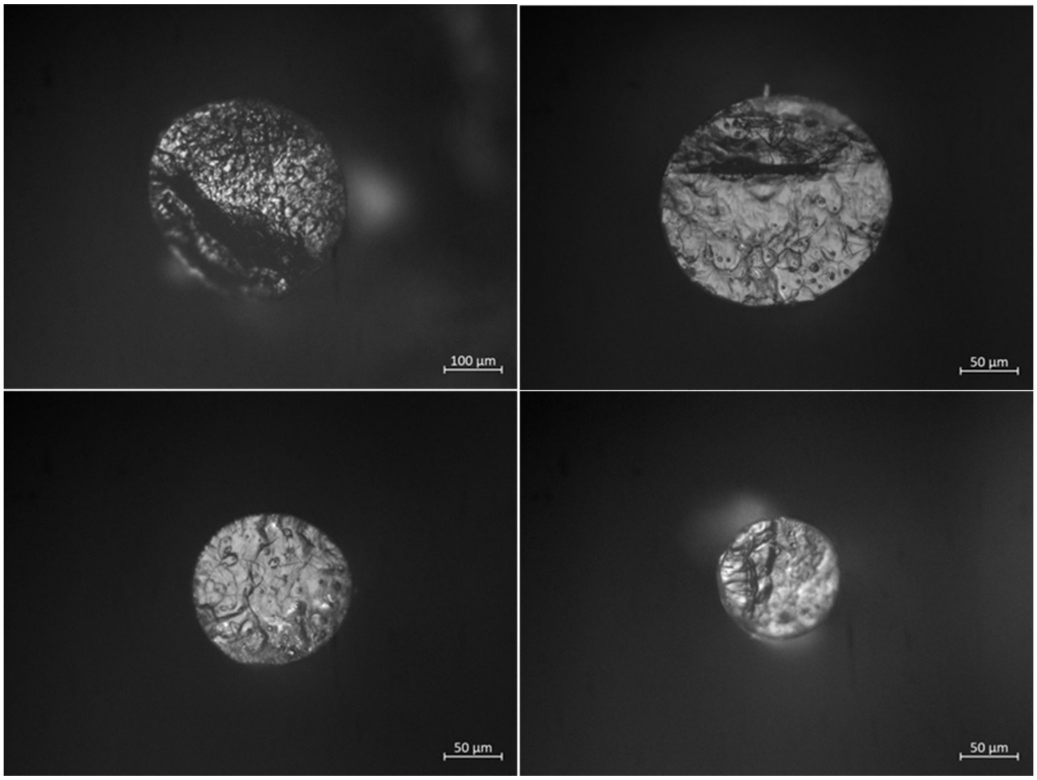

Figure S24. Cross-sectional images of filaments printed at 1, 6, 10, and 15 mm s^{-1}.

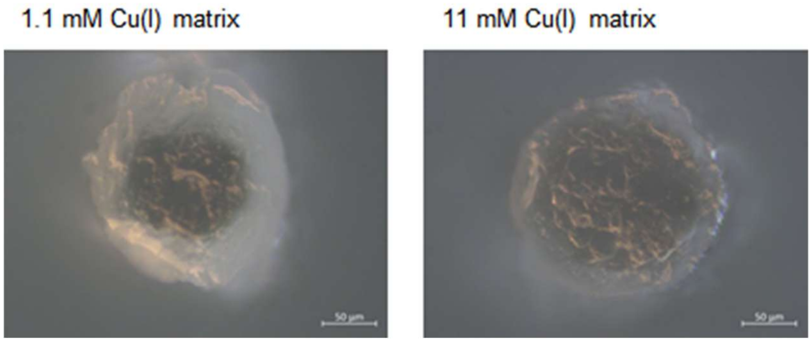

Figure S25. Optical microscope images of printed CNT composite filaments (1 wt% MWCNT) under two different curing bath conditions: low activator concentration (1.1 mM Cu(I), left) and high activator concentration (11 mM Cu(I), right).

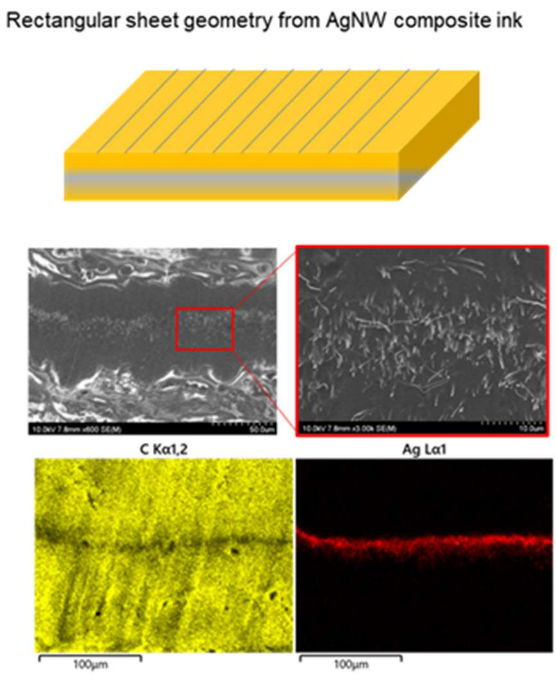

Figure S26. Schematic and energy-dispersive X-ray spectroscopy (EDS) mapping of printed AgNW composite ink in rectangular sheet geometry overlayed on the zoomed-in region of the SEM image of the printed structure.

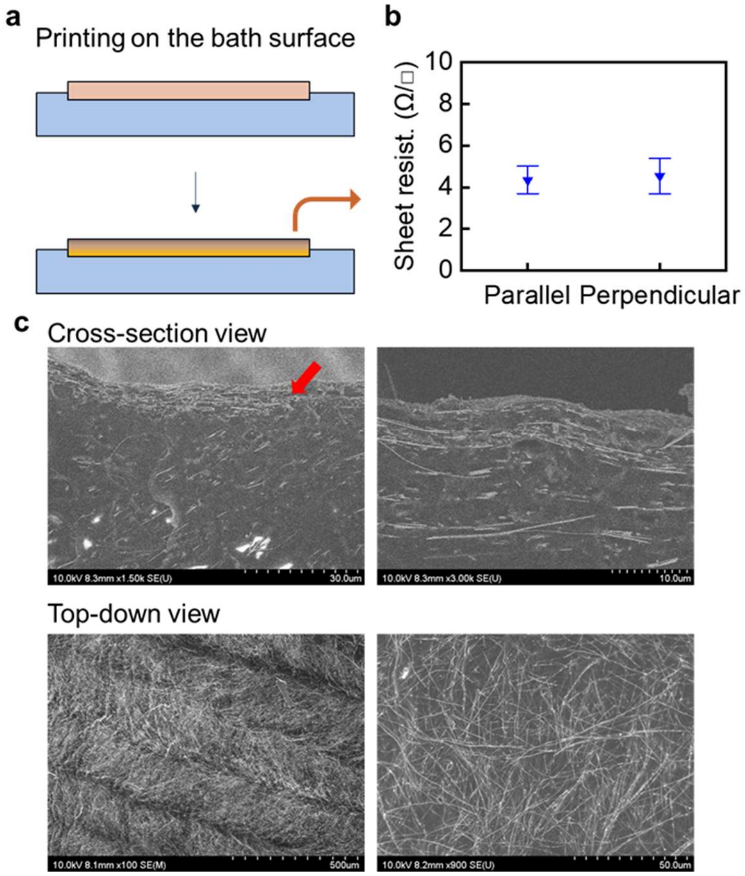

Figure S27. (a) Schematic of AgNW-rich surface obtained by depositing the composite ink onto the top of the matrix rather than embedding it. (b) Sheet resistance measurements in parallel and perpendicular directions. (c) SEM images showing the cross-sectional and top-down views.

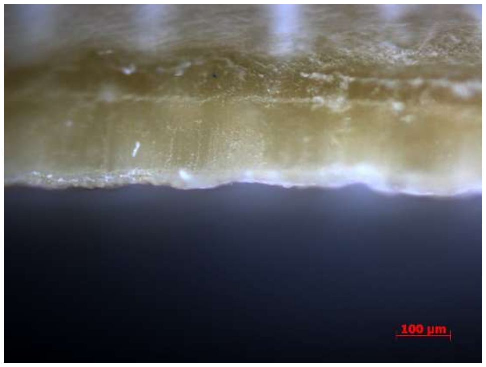

Figure S28. Cross-sectional optical image of a printed sheet taken perpendicular to the printing direction.

3. Supporting Videos

Supporting Video S1. Embedded 3D printing and spontaneous curing process in PG matrix and PEG matrix. The video is presented at 10× speed, with a scale bar of 5 mm.

Supporting Video S2. Effect of ink and matrix viscosity on printed filament morphology. The video is presented at 1× speed, with a scale bar of 5 mm.

Supporting Video S3. Snap-through buckling video using a shallow spherical shell structure fabricated via EMB3D.

References

[1] L. M. Dean, Q. Wu, O. Alshangiti, J. S. Moore, N. R. Sottos, *ACS Macro Lett.* **2020**, *9*, 819.
